# *Z*-selective hydrosilylation of terminal alkynes catalyzed by a bench-stable manganese alkyl complex

**DOI:** 10.1007/s00706-026-03510-0

**Published:** 2026-08-15

**Authors:** Daniel P. Zobernig, Karl Kirchner

**Affiliations:** Institute of Applied Synthetic Chemistry, TU Wien, Getreidemarkt 9/163-AC, 1060 Vienna, Austria

**Keywords:** N-heterocyclic carbine, Manganese, Hydrosilylation, Alkynes

## Abstract

A stereo- and regioselective Mn(I)-catalyzed hydrosilylation of terminal alkynes is described. The hydrosilylation reaction selectively yields the thermodynamically less stable (*Z*)-alkenyl silane. A wide variety of aromatic and aliphatic alkynes were efficiently and selectively hydrosilylated. The reactions proceed with catalyst loadings of 1–2 mol% at 60 °C in THF without the need of any additives. The active precatalyst is the bench-stable alkyl Mn(I) complex *fac*-[Mn(PC-*i*Pr)(CO)_3_(CH_2_CH_2_CH_3_)].

**Graphical abstract:**

**Figure:**
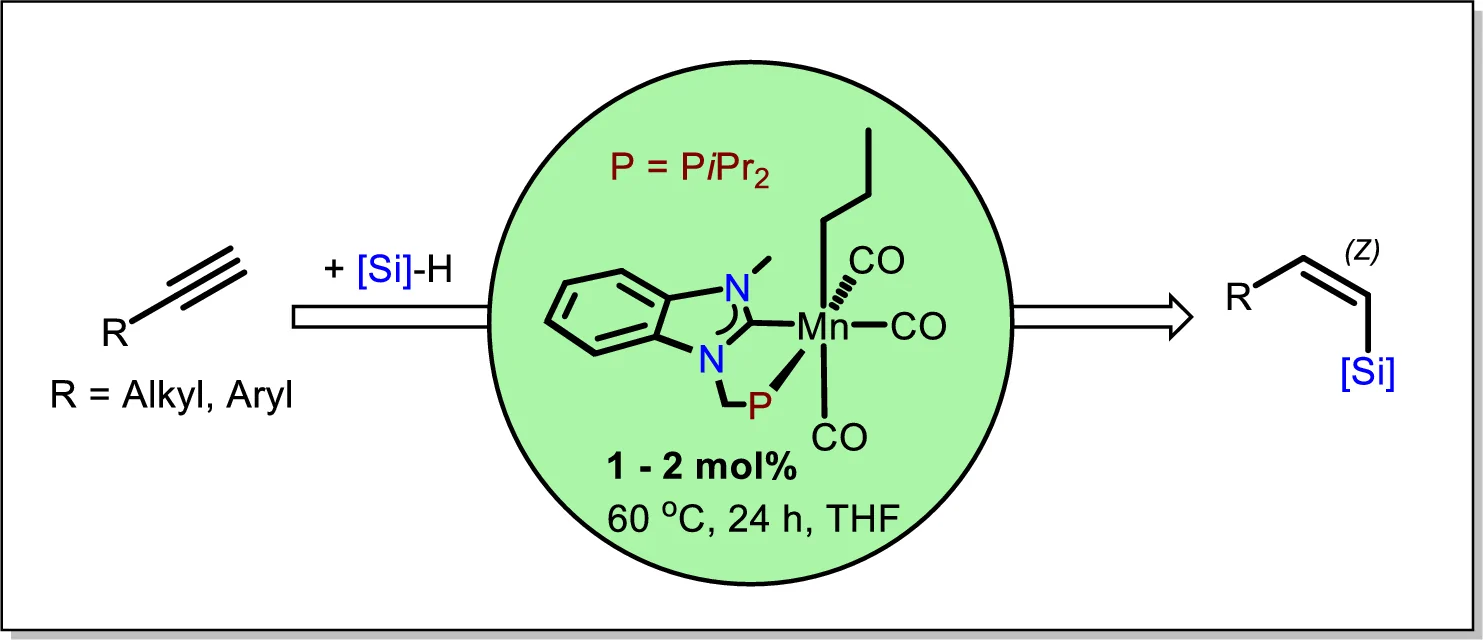

**Supplementary Information:**

The online version contains supplementary material available at https://doi.org/10.1007/s00706-026-03510-0.

## Introduction

A straightforward approach to obtain vinylsilanes is the catalytic hydrosilylation of alkynes using transition metal complexes [1–7]. An important issue is to control the regio- and stereoselectivity as these reactions often afford mixtures of regio- and stereoisomers such as *α*-, *β*-(*Z*)-, and *β*-(*E*)-vinylsilanes with the *β*-isomers being the results of an *anti*-Markovnikov addition of a Si–H bond to an alkyne moiety.

Moreover, the development of sustainable, efficient, and selective syntheses is one of the fundamental research goals in modern chemistry. In this context, it is important to perform reactions under catalytic conditions and to replace precious metal catalysts by earth abundant non-precious metal catalysts. In particular base metals such as iron or manganese constitute promising candidates as these belong to the most abundant metals in the Earth’s crust, are inexpensive, and exhibit a low environmental impact.

Accordingly, taking into account the synthetic usefulness of vinylsilanes, the hydrosilylation of alkynes has become an important subject in base metal catalysis [4, 8–13] including rare reports with Mn [14–17]. The first reported was the Wang and co-workers, who used 5 mol% of [Mn(CO)_5_Br] and 20 mol% of AsPh_3_ to hydrosilylate internal alkynes to the corresponding (*E*)-alkenylsilanes at 150 °C [14]. They could also (*Z*)-selectively hydrosilylate internal and terminal alkynes with 10 mol% [Mn_2_(CO)_10_] and 20 mol% lauroyl peroxide as radical initiator at 120 °C. In 2019, Zhang, Zhang, and co-workers utilized 10 mol% [Mn_2_(CO)_10_] and a blue LED to hydrosilylate both internal and terminal alkynes (*Z)*-selectively [15]. Detailed theoretical (DFT) by studies by Li and co-workers provide a mechanism and origin of the stereoselectivity of manganese-catalyzed hydrosilylation of alkynes in both mononuclear [Mn(CO)_5_Br] and binuclear [Mn_2_(CO)_10_]-catalyzed cycles [16]. Finally, Bagh and co-workers described in 2023 a cationic Mn(I) complex bearing a tridentate ligand with an N-heterocylic carbon at its center and two pyridine arms as side donors as efficient catalyst for the formation of alkenylsilanes with high yields and with good selectivity towards the (*Z*)-isomer [17] (see Scheme 1).

**Figure:**
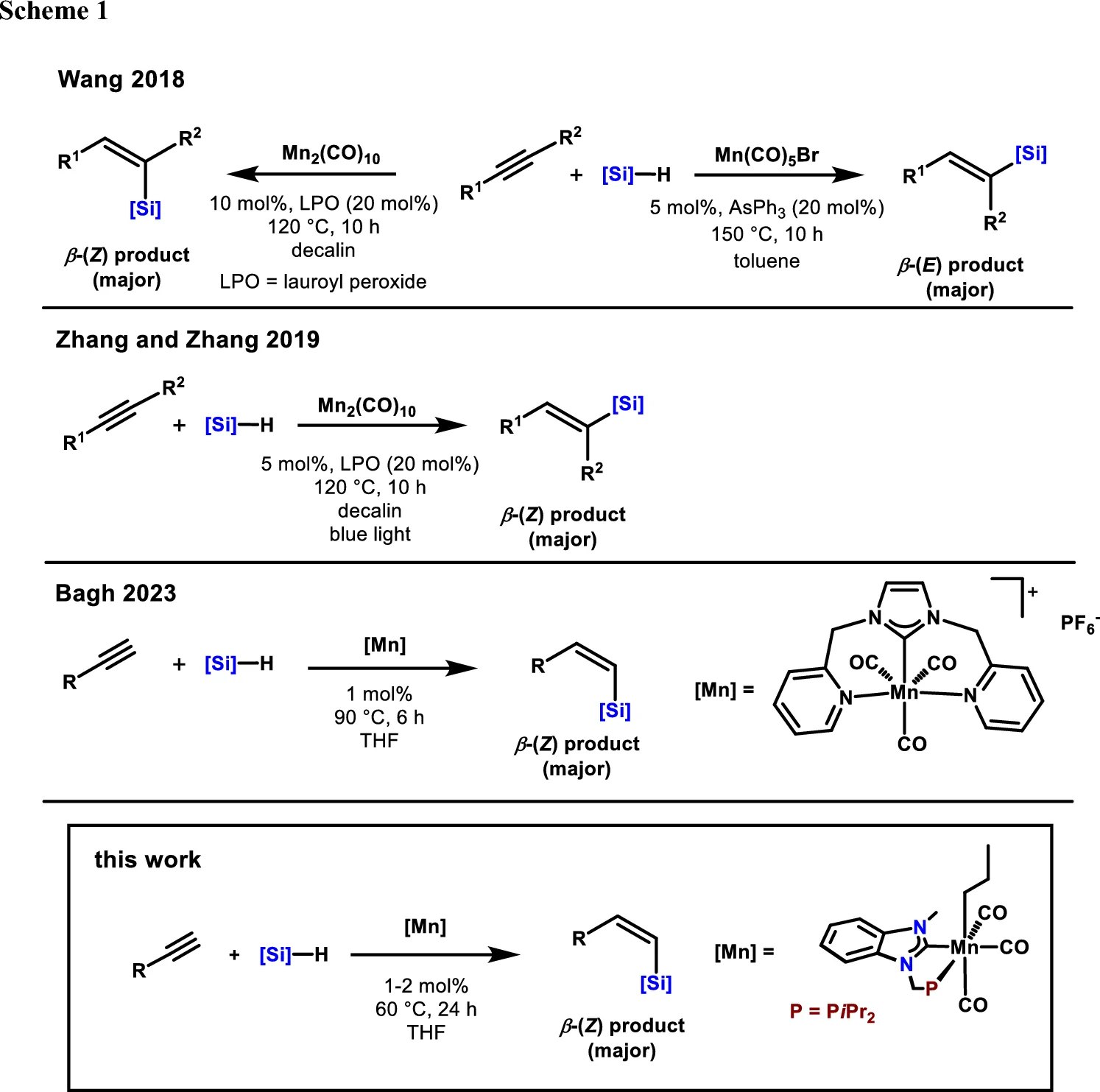

Here we report on the bench-stable Mn(I) alkyl complex *fac*-[Mn(PC-*i*Pr)(CO)_3_(CH_2_CH_2_CH_3_)] (**1**) [18] as pre-catalyst for the hydrosilylation of terminal alkenes to afford in a controllable fashion thermodynamically less stable *β*-(*Z*)-alkenyl silanes.

## Results and discussion

The catalytic performance of **1** as hydrosilylation pre-catalyst was first investigated with phenylacetylene as model substrate and various primary, secondary, and tertiary silanes at 60 °C. Optimization experiments are depicted in Table 1. With catalyst loading of 1 mol% in THF as a solvent utilizing SiH_3_Hex, SiH_2_Et_2_, SiH_2_Ph_2_, SiH_2_MePh, and SiHMe_2_Ph mixtures of *β*-(*Z*)- (**3**) and *α*-products (**4**) were observed with conversions ranging from 19 to 85% (Table 1, entries 1–5). There was no evidence for the formation of the *β*-(*E*)-product (**2**). The highest conversions and best **2**:**3**:**4** ratios were achieved with the secondary silanes SiH_2_Ph_2_ and SiH_2_MePh being 0:99:1 and 0:97:3, respectively.

**Table 1:** Optimization reactions for the manganese-catalyzed hydrosilylation of phenylacetylene


Entry	x / mol%	[Si]	y / equiv	Solvent	Conversion / %	**2**/**3**/**4**
1	1	SiH_3_Hex	1.1	THF	19	0/72/28
2	1	SiH_2_Et_2_	1.1	THF	47	0/77/23
3	1	SiH_2_Ph_2_	1.1	THF	75	0/99/1
4	1	SiH_2_MePh	1.1	THF	85	0/97/3
5	1	SiHMe_2_Ph	1.1	THF	52	0/99/1
6	1	SiH_2_MePh	1.1	Neat	84	1/89/10
7	1	SiH_2_MePh	1.1	Toluene	57	0/96/4
8	1	SiH_2_MePh	1.1	DCE	63	0/94/6
**9**	**2**	**SiH**_**2**_**MePh**	**1.1**	**THF**	** > 99**	**0/94/6**
**10**	**2**	**SiH**_**2**_**Ph**_**2**_	**1.1**	**THF**	** > 99**	**0/99/1**

Reaction conditions: phenylacetylene (25 mm^3^, 0.23 mmol, 1 equiv.), silane (0.24–0.27 mmol, 1.05–1.2 equiv.), **1** (1–2 mg, 2.3–4.6 µmol, 1–2 mol%), 0.5 cm^3^ solvent, 60 °C, 24 h, conversion and isomer ratio determined by GC–MS

Under neat conditions with SiH_2_MePh, the conversion was still high, but the **2**:**3**:**4** ratio turned out to be slightly worse being 1:89:10 (Table 1, entry 6). With the same silane in toluene and DCE the conversion dropped from 85% in THF to 57 and 63%, respectively (Table 1, entries 7 and 8). By raising the catalyst loading from 1 to 2 mol% in THF with SiH_2_Ph_2_ or SiH_2_MePh the conversions were essentially quantitative (Table 1, entries 9 and 10). Finally, in the absence of catalyst no reaction takes place.

Having established reasonably good reaction conditions (2 mol % catalyst, 60 °C, THF), the scope and limitations for the hydrosilyation of terminal alkynes were examined with the silanes SiH_2_Ph_2_ and SiH_2_MePh (Table 2). Several aromatic and aliphatic alkynes with both electron-withdrawing and electron-donating moieties were tested. With phenylacetylene full conversion was achieved with a selectivity of 97% and 90% of the *β*-(*Z*)-isomer, respectively (**3a**, **3b**). The **3**:**4** rations were 97:3 and 90:10, respectively.

**Table 2:**
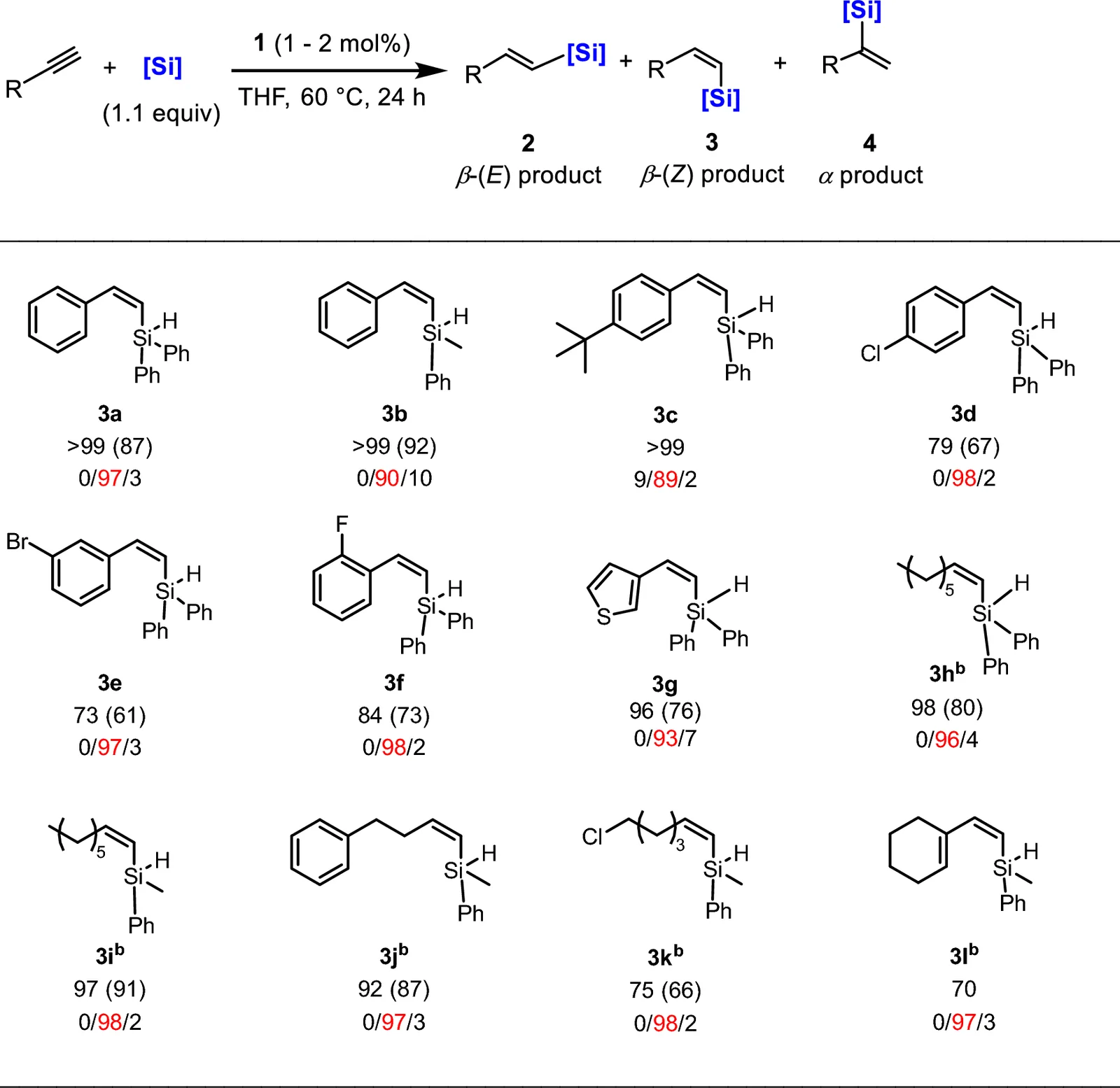
Hydrosilylation of terminal alkynes

Reaction conditions: alkyne (0.50 mmol, 1 equiv.), silane (0.55 mmol, 1.1 equiv.), **1** (2 mol%), 1 cm^3^ THF, 60 °C, 24 h, conversion and product ratio determined by GC–MS, isolated yield in parentheses, product ratio given as **2**/**3**/**4**. ^b^ **1** (1 mol%)

Electron-donating and electron-withdrawing groups in *para*-position were well tolerated (**3c**, **3d**). Aromatic substrates with substituents at the *meta*- and *ortho*-position were also tested and exhibited very good conversions with excellent selectivity towards the corresponding *β*-(*Z*)-alkenylsilanes (**3e**, **3f**). In this context, 3-ethynylthiophene was also successfully hydrosilylated with an excellent conversion of 96% and a 93:7 ratio of **3**:**4** (**3g**). Aliphatic alkynes were hydrosilylated to *β*-(*Z*)-alkenylsilanes even with a catalyst loading reduced to 1 mol% (**3h**, **3i**). Functional groups such as phenyl and chloro groups were also well tolerated and resulted in very good conversions together with excellent selectivities (**3j**, **3k**). Moreover, 1-ethynylcyclohexene was successfully hydrosilylated to the corresponding *β*-(*Z*)-alkenylsilane albeit with lower conversion of 70% but an excellent **3**:**4** ratio of 97:3 (**3l**).

In addition, in order to get some mechanistic insights of the catalytic reaction deuterium labeling studies were conducted. Phenylacetylene-*d*_*1*_ and 1-octyne-*d*_*1*_ were reacted with SiH_2_Ph_2_ under the optimized reaction conditions with 2 mol% of catalyst **1** (Scheme 2). The deuterium distribution of the products was analyzed by ^1^H NMR spectroscopy. For phenylacetylene there was about 30% of deuterium incorporation into the Si–H group of the vinylsilane which may be indicative of some C–D bond activation of the phenylacetylene-*d*_*1*_. Furthermore, the β-*E* product showed no deuterium at the benzylic position but still 70% of D at the terminal carbon atom. In the case of 1-octyne deuterium incorporation into the Si–H group was negligible and no deuterium was found at the internal vinyl carbon atom and basically 100% D remained at the terminal carbon atom, thus, excluding any C–D bond activation step. The slightly different behavior of aromatic terminal alkynes may be due to their lower p*K*_a_ value as compared to aliphatic ones. The acidity of the terminal C–H bond of aromatic and aliphatic alkynes is quite different (p*K*_a_ (aromatic) ≈ 23, p*K*_a_ (aliphatic) ≈ 25)) [19].

**Figure:**
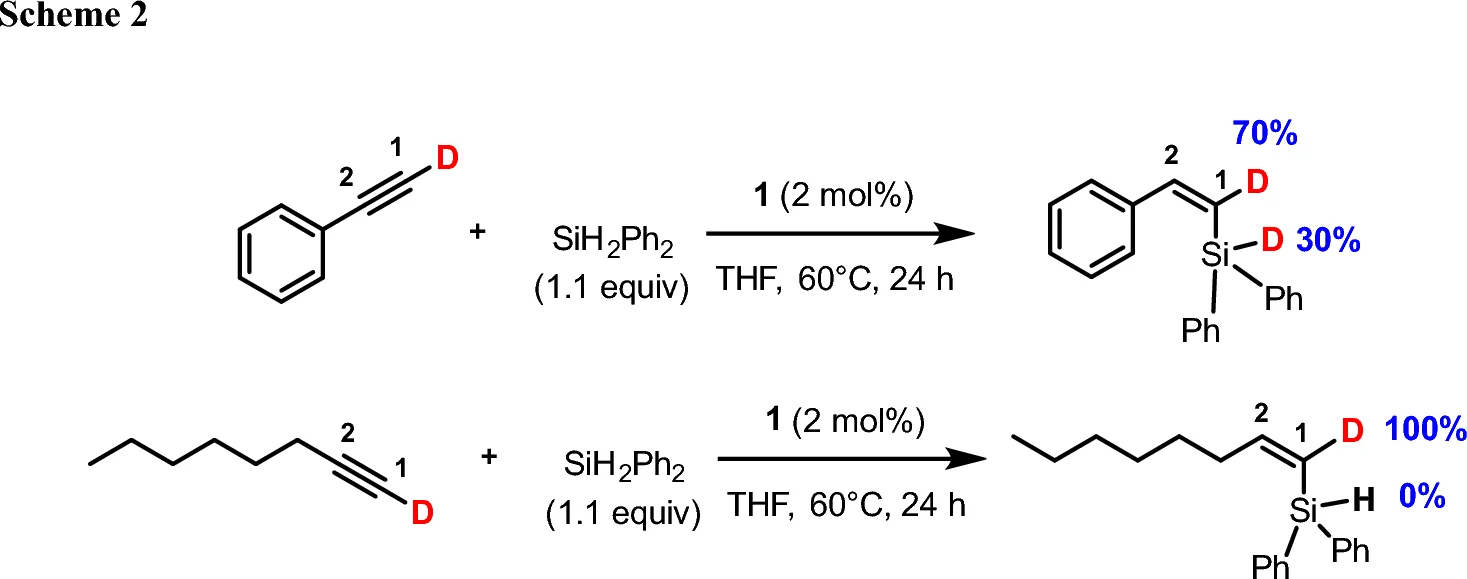

It may be noted that we previously observed for the hydroboration of terminal alkynes with Mn(I) alkyl catalysts that in the case of phenylacetylene-*d*_*1*_ the deuterium ended up exclusively at the benzylic position indicating that C–D bond cleavage took place [20]. In contrast, with octyne-*d*_*1*_ no deuterium migration occurred. These findings reveal that two diverging reaction pathways depending on the acidity of the C–H bond of the alkyne can occur. In the present case of HS, this is not the case as we did not observe D incorporation at carbon 2.

Based on deuterium labeling studies it is clear that C–H bond activation of the terminal alkynes forming for instance an acetylide species is not taking place. A simplified mechanism is proposed in Scheme 3. We assume that the actual catalyst is the silyl species **A** (formed from **1** involving several as yet not completely understood initiation steps [21]) which forms upon insertion of an alkyne into the Mn–Si bond the *E*-vinyl intermediate **C**. This species may undergo an Ojima–Crabtree type rearrangement [22, 23]. This rearrangement effectively rotates and rearranges metal-vinyl intermediate **C** to afford via intermediates **D** or **E** the *Z*-vinyl intermediate **F** ultimately yielding *β*-(*Z*)-vinylsilanes. This was proposed recently also for manganese and cobalt catalyzed *Z*-selective hydrosilylations of terminal alkynes [16, 24, 25].

**Figure:**
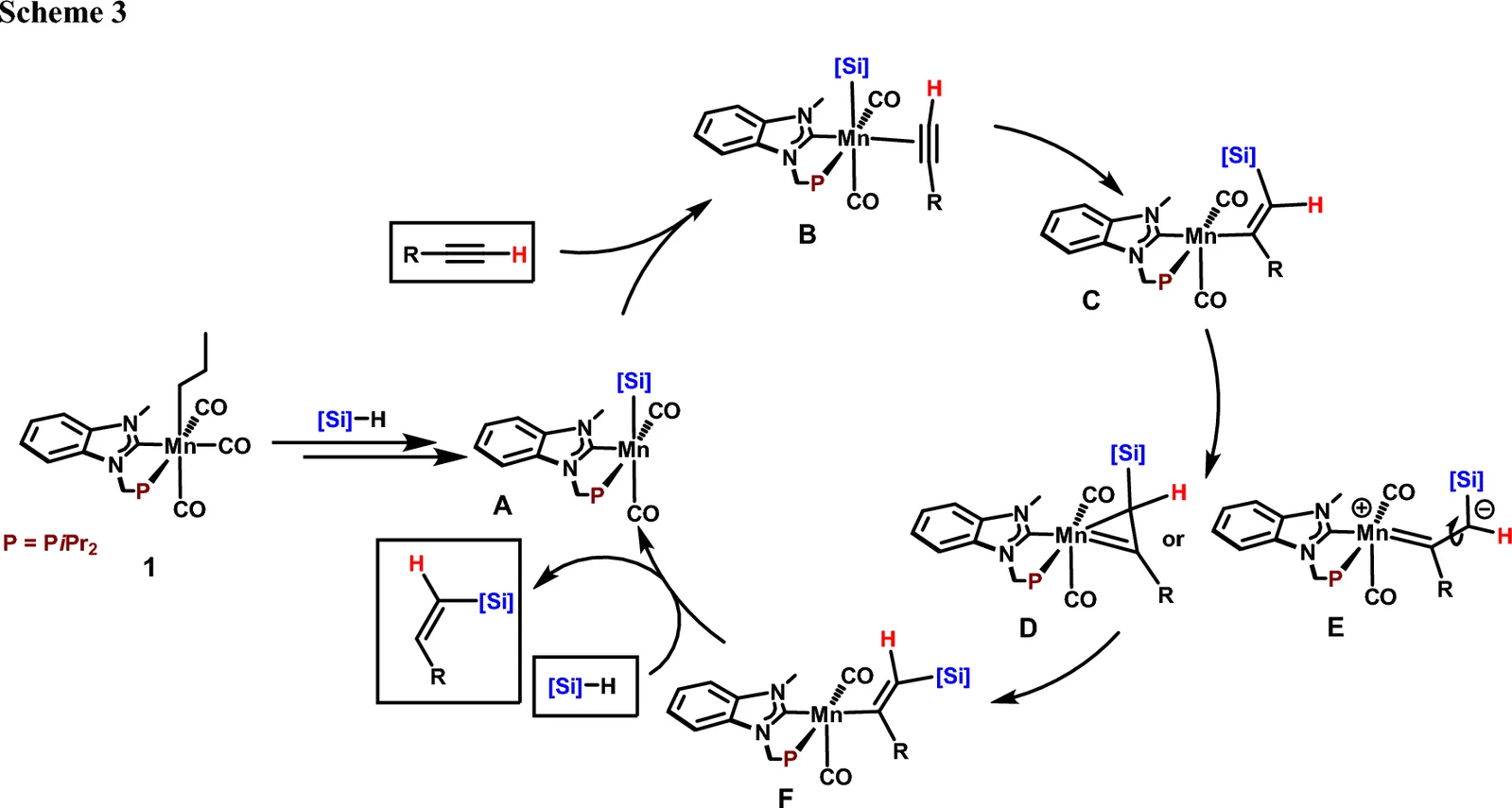

## Conclusion

In sum, we have developed a Mn(I)-catalyzed *Z*-selective *anti*-Markovnikov hydrosilylation of terminal alkynes with several primary, secondary, and tertiary silanes. Pre-catalyst is the bench-stable alkyl Mn(I) complex *fac*-[Mn(PC-*i*Pr)(CO)_3_(CH_2_CH_2_CH_3_)]. A range of aromatic and aliphatic alkynes underwent this reaction to afford *β*-(*Z*)-vinylsilanes in high yields with excellent stereoselectivities. The thermodynamically more stable *β*-(*E*)-vinylsilanes were in most cases not detected. Based on deuterium labeling studies it was shown that C–H bond activation of the terminal alkynes are not taking place. Instead, we proposed that an *E*-alkenyl intermediate undergoes an Ojima–Crabtree type rearrangement. Due to steric interference between the bulky silyl group and the metal-fragment, this rearrangement effectively rotates and rearranges the metal-(*E*)-alkenyl intermediate and ultimately yields the *β*-(*Z*)-vinylsilane products.

## Experimental

All reactions were performed under an inert atmosphere of argon by using Schlenk techniques or in a MBraun inert-gas glovebox. The solvents were purified according to standard procedures [26]. All substrates were purchased from Sigma-Aldrich, Acros Organics, TCI, or BLDpharm and used as purchased without further purification. Complex *fac*-[Mn(PC-*i*Pr)(CO)_3_(CH_2_CH_2_CH_3_)] (**1**) was synthesiszed according to the literature [18]. The deuterated solvents were purchased from Eurisotope and dried over 3 Å molecular sieves. ^1^H, ^13^C{^1^H}, and ^29^Si{^1^H} NMR spectra were recorded on Bruker AVANCE-400, AVANCE-NEO-400 and AVANCE-600 spectrometers. ^1^H and ^13^C{^1^H} NMR spectra were referenced internally to residual protio-solvent, and solvent resonances, respectively, and are reported relative to tetramethylsilane (*δ* = 0 ppm), ^29^Si{^1^H} NMR spectra were referenced externally to tetramethylsilane (*δ* = 0 ppm). Preparative flash column chromatography was conducted manually using glass columns packed with silica gel 60 (Merck, 40–63 µm).

GC–MS analyses were conducted on a ISQ LT Single quadrupole MS (Thermo Fisher) directly interfaced to a TRACE 1300 Gas Chromatographic systems (Thermo Fisher), using a Rxi-5Sil MS (30 m, 0.25 mm ID) cross-bonded dimethyl polysiloxane capillary column at a carrier flow of He 1.5 cm^3^/min.

### General procedure for hydrosilylation of alkynes

Inside an argon flushed glovebox, a screw cap vial (8 cm^3^) was charged with catalyst (2.2–4.4 mg, 1–2 mol%), alkyne (0.5 mmol, 1 equiv.), silane (0.55 mmol, 1.1 equiv.) and THF (1 cm^3^) in this order. A stirring bar was added, the vial was closed, transferred out of the glovebox and was stirred for a specific time at a given temperature. Afterwards the reaction mixture was allowed to reach room temperature and exposed to air to quench the catalyst. 2 mm^3^ of the sample was analyzed via GC–MS. The solvent was subsequently removed in vacuo and the crude product was purified either via column chromatography or a thin pad of silica with specified eluent. The products were characterized by ^1^H, ^13^C{^1^H}, and ^29^Si{^1^H} NMR spectroscopy or GC–MS if the products could not be isolated in pure form.

### Deuteration experiment with 1-octyne-***d***_***1***_, phenylacetylene-***d***_***1***_, and diphenylsilane

Inside an argon flushed glovebox, a screw cap vial (8 cm^3^) was charged with **1** (2 mg, 2 mol%), either 1-octyne-*d*_*1*_ (33 mm^3^, 0.23 mmol, 1.0 equiv.) or phenylacetylene-*d*_*1*_ (25 mm^3^, 0.23 mmol, 1.0 equiv.), diphenylsilane (46 mm^3^, 0.25 mmol, 1.1 equiv.) and THF (0.5 cm^3^) in this order. A stirring-bar was added, the vial was closed, transferred out of the glovebox and was stirred for 24 h at 60 °C. Afterwards the reaction mixture was allowed to reach room temperature and exposed to air to quench the catalyst. The reaction mixture was characterized with ^1^H spectroscopy to determine the hydrogen and deuterium incorporation.

## Supplementary Information

Below is the link to the electronic supplementary material.Supplementary file1 (PDF 9689 KB)

## Data Availability

All data that support the findings of this study are available in the published article and/or the supplementary material to this article.
