# Supplementary material for: Z-selective hydrosilylation of terminal alkynes catalyzed by a bench-stable manganese alkyl complex
Source: Monatsh Chem. 2026 Aug 15;157(9):1221–6. doi: 10.1007/s00706-026-03510-0 (PMC13529888; doi:10.1007/s00706-026-03510-0)
Supplement: Supplementary file 1 [file 706_2026_3510_MOESM1_ESM.pdf]

Z-Selective Hydrosilylation of Terminal Alkynes Catalyzed by a Bench-Stable Manganese Alkyl Complex

Daniel P. Zobernig and Karl Kirchner*

Institute of Applied Synthetic Chemistry, TU Wien, Getreidemarkt 9/163-AC, A-1060 Wien, Austria.

Supporting Information

Characterization of Organic Products 2
NMR Spectra 4

Characterization of Organic Products

Diphenyl-[(1*Z*)-2-phenylethen-1-yl]silane (3a)

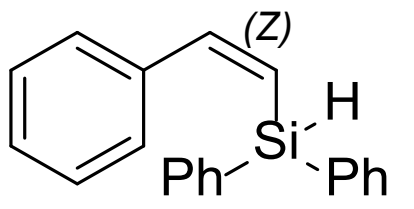

phenylacetylene (55 µL, 0.5 mmol, 1 equiv.); diphenylsilane (102 µL, 0.55 mmol, 1.1 equiv.); catalyst (4.4 mg, 2 mol%); eluent for column chromatography: petroleum ether; yield: 125 mg (87%) of a colorless oil. ^{1}H NMR (400 MHz, $CDCl_3$): δ = 7.51 (d, *J* = 15.0 Hz, 1H), 7.47 – 7.44 (m, 4H), 7.27 – 7.21 (m, 8H), 7.15 – 7.10 (m, 3H), 6.03 (dd, *J* = 14.9, 6.1 Hz, 1H), 5.16 (d, *J* = 6.1 Hz, 1H) ppm. ^{13}C{^{1}H} NMR (101 MHz, $CDCl_3$): δ = 150.3, 138.9, 135.4, 134.6, 129.8, 128.7, 128.2, 128.2, 128.1, 124.6 ppm. ^{29}Si{^{1}H} NMR (79 MHz, $CDCl_3$): δ = -28.7 ppm.

Methylphenyl-[(1*Z*)-2-phenylethen-1-yl]silane (3b)

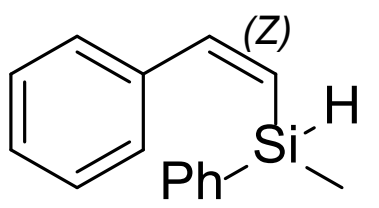

phenylacetylene (55 µL, 0.5 mmol, 1 equiv.); methylphenylsilane (76 µL, 0.55 mmol, 1.1 equiv.); catalyst (4.4 mg, 2 mol%); eluent for thin pad of silica: petroleum ether; yield: 103 mg (92%) of a colorless oil (only main isomer characterized). ^{1}H NMR (400 MHz, $CDCl_3$): δ = 7.61 – 7.56 (m, 2H), 7.52 (d, *J* = 14.9 Hz, 1H), 7.39 – 7.25 (m, 8H), 5.99 (dd, *J* = 15.0, 5.5 Hz, 1H), 4.88 – 4.81 (m, 1H), 0.41 (d, *J* = 3.8 Hz, 3H) ppm. ^{13}C{^{1}H} NMR (101 MHz, $CDCl_3$): δ = 149.1, 139.2, 136.3, 134.6, 129.5, 128.6, 128.2, 128.1, 127.9, 126.8, -3.8 ppm. ^{29}Si{^{1}H} NMR (79 MHz, $CDCl_3$): δ = -28.1 ppm.

[(1*Z*)-2-(4-*tert*-Butylphenyl)ethen-1-yl]diphenylsilane (3c)

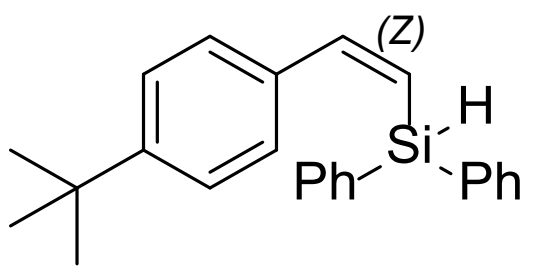

4-*tert*-butylphenylacetylene (90 µL, 0.5 mmol, 1 equiv.); diphenylsilane (102 µL, 0.55 mmol, 1.1 equiv.); catalyst (4.4 mg, 2 mol%); conversion: >99% (product was not isolated as it isomerized to the *E*-isomer).

[(1*Z*)-2-(4-Chlorophenyl)ethen-1-yl]diphenylsilan (3d)

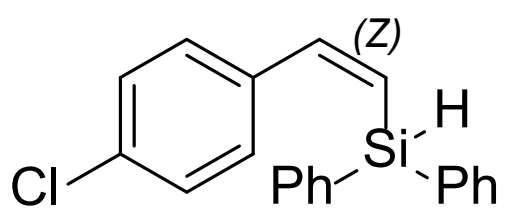

1-chloro-4-ethynylbenzene (68 mg, 0.5 mmol, 1 equiv.); diphenylsilane (102 µL, 0.55 mmol, 1.1 equiv.); catalyst (4.4 mg, 2 mol%); eluent for column chromatography: petroleum ether; yield: 101 mg (67%) of a colorless solid. ^{1}H NMR (400 MHz, $CDCl_3$): δ = 7.61 – 7.55 (m, 5H), 7.44 – 7.33 (m, 6H), 7.31 – 7.26 (m, 2H), 7.25 – 7.20 (m, 2H), 6.21 (dd, *J* = 14.9, 6.0 Hz, 1H), 5.27 (d, *J* = 6.0 Hz, 1H). ^{13}C{^{1}H} NMR (101 MHz, $CDCl_3$): δ = 148.9, 137.3, 135.4, 134.2, 134.0, 129.9, 129.9, 128.4, 128.3, 125.6 ppm. ^{29}Si{^{1}H} NMR (79 MHz, $CDCl_3$): δ = -28.9 ppm.

[(1*Z*)-2-(3-Bromophenyl)ethen-1-yl]diphenylsilan (3e)

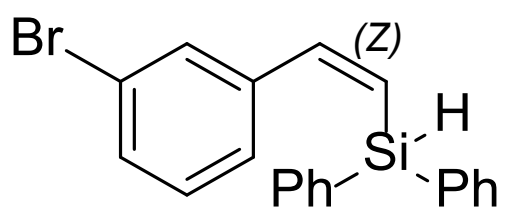

3-bromophenylacetylene (62 µL, 0.5 mmol, 1 equiv.); diphenylsilane (102 µL, 0.55 mmol, 1.1 equiv.); catalyst (4.4 mg, 2 mol%); eluent for column chromatography: petroleum ether; yield: 112 mg (61%) of a yellow oil. ^{1}H NMR (400 MHz, $CDCl_3$): δ = 7.62 – 7.53 (m, 5H), 7.51 – 7.46 (m, 1H), 7.43 – 7.32 (m, 7H), 7.27 – 7.24 (m, 1H), 7.11 (t, *J* = 7.8 Hz, 1H), 6.25 (dd, *J* = 14.9, 5.8 Hz, 1H), 5.26 (d, *J* = 5.8 Hz, 1H) ppm. ^{13}C{^{1}H} NMR (101 MHz, $CDCl_3$): δ = 148.6, 140.9, 135.4, 134.1, 131.6, 130.9, 129.9, 129.7, 128.3, 127.1, 126.8, 122.3 ppm. ^{29}Si{^{1}H} NMR (79 MHz, $CDCl_3$): δ = -28.9 ppm.

[(1*Z*)-2-(2-Fluorophenyl)ethen-1-yl]diphenylsilan (3f)

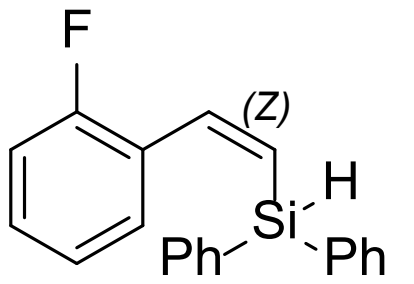

1-fluoro-2-ethynylbenzene (59 µL, 0.5 mmol, 1 equiv.); diphenylsilane (102 µL, 0.55 mmol, 1.1 equiv.); catalyst (4.4 mg, 2 mol%); eluent for column chromatography: petroleum ether; yield: 112 mg (73%) of a colorless oil. ^{1}H NMR (400 MHz, $CDCl_3$): δ = 7.78 – 7.66 (m, 1H), 7.61 – 7.53 (m, 4H), 7.43 – 7.30 (m, 7H), 7.25 – 7.18 (m, 1H), 7.06 – 6.93 (m, 2H), 6.32 (dd, *J* = 15.0, 6.0 Hz, 1H), 5.20 (dd, *J* = 6.0, 1.9 Hz, 1H) ppm. ^{13}C{^{1}H} NMR (101 MHz, $CDCl_3$): δ: 160.4 (d, *J* = 248.3 Hz), 142.6 (d, *J* = 3.8 Hz), 135.4, 134.2, 130.1 (d, *J* = 3.1 Hz), 129.9, 129.8, 128.2, 127.3, 126.8 (d, *J* = 13.6 Hz), 123.6 (d, *J* = 3.6 Hz), 115.3 (d, *J* = 21.7 Hz) ppm. ^{29}Si{^{1}H} NMR (79 MHz, $CDCl_3$): δ = -28.7 ppm.

Diphenyl-[(1*Z*)-2-(3-thienyl)ethen-1-yl]silan (3g)

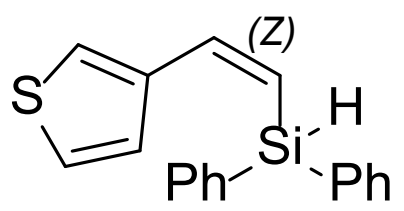

3-ethynylthiophene (49 µL, 0.5 mmol, 1 equiv.); diphenylsilane (102 µL, 0.55 mmol, 1.1 equiv.); catalyst (4.4 mg, 2 mol%); eluent for column chromatography: petroleum ether; yield: 124 mg (76%) of a colorless oil. ^{1}H NMR (400 MHz, $CDCl_3$): δ = 7.66 – 7.52 (m, 5H), 7.44 – 7.34 (m, 6H), 7.29 – 7.26 (m, 1H), 7.23 – 7.15 (m, 2H), 6.07 (dd, *J* = 15.0, 5.8 Hz, 1H), 5.39 (d, *J* = 5.9 Hz, 1H) ppm. ^{13}C{^{1}H} NMR (101 MHz, $CDCl_3$): δ = 143.7, 141.2, 135.4, 134.3, 129.8, 128.3, 128.1, 125.5, 125.3, 123.0 ppm. ^{29}Si{^{1}H} NMR (79 MHz, $CDCl_3$): δ = -28.8 ppm.

[(1*Z*)-1-Octen-1-yl]diphenylsilane (3h)

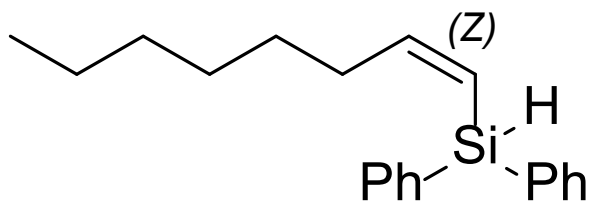

1-Octyne (73 µL, 0.5 mmol, 1 equiv.); diphenylsilane (102 µL, 0.55 mmol, 1.1 equiv.); catalyst (2.2 mg, 1 mol%); eluent for column chromatography: petroleum ether; yield: 117 mg (80%; 11% diphenylsilane as impurity) of a colorless oil ^{1}H NMR (400 MHz, $CDCl_3$): δ = 7.62 – 7.56 (m, 4H), 7.43 – 7.31 (m, 6H), 6.67 (dt, *J* = 13.8, 7.4 Hz, 1H), 5.84 (ddt, *J* = 13.8, 5.4, 1.3 Hz, 1H), 5.27 (d, *J* = 5.3 Hz, 1H), 2.20 (q, *J* = 7.4 Hz, 2H), 1.40 – 1.30 (m, 2H), 1.29 – 1.12 (m, 6H), 0.85 (t, *J* = 7.0 Hz, 3H) ppm. ^{13}C{^{1}H} NMR (101 MHz, $CDCl_3$) δ: 153.9, 135.4, 134.8, 129.6, 128.1, 121.5, 33.9, 31.8, 29.4, 29.0, 22.7, 14.2 ppm. ^{29}Si{^{1}H} NMR (79 MHz, $CDCl_3$): δ = -31.6 ppm. This spectroscopic data corresponds to previously reported findings.[8]

Methyl-[(1*Z*)-1-octen-1-yl]phenylsilane (3i)

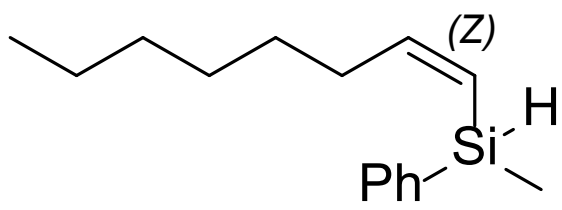

1-Octyne (73 µL, 0.5 mmol, 1 equiv.); methylphenylsilane (76 µL, 0.55 mmol, 1.1 equiv.); catalyst (2.2 mg, 1 mol%); eluent for thin pad of silica: petroleum ether; yield: 106 mg (91%) of a colorless oil. ^{1}H NMR (400 MHz, $CDCl_3$): δ = 7.59 – 7.53 (m, 2H), 7.38 – 7.32 (m, 3H), 6.52 (dt, *J* = 13.8, 7.4 Hz, 1H), 5.63 (ddt, *J* = 13.8, 5.1, 1.3 Hz, 1H), 4.77 – 4.71 (m, 1H), 2.24 – 2.13 (m, 2H), 1.42 – 1.31 (m, 3H), 1.31 – 1.18 (m, 6H), 0.87 (t, *J* = 6.9 Hz, 3H), 0.42 (d, *J* = 3.9 Hz, 3H) ppm. ^{13}C{^{1}H} NMR (101 MHz, $CDCl_3$): δ = 152.4, 136.7, 134.6, 129.3, 128.0, 123.6, 33.7, 31.8, 29.5, 29.0, 22.7, 14.2, -4.0 ppm. ^{29}Si{^{1}H} NMR (79 MHz, $CDCl_3$): δ = -30.8 ppm.

Methylphenyl-[(1*Z*)-4-phenyl-1-buten-1-yl]silane (3j)

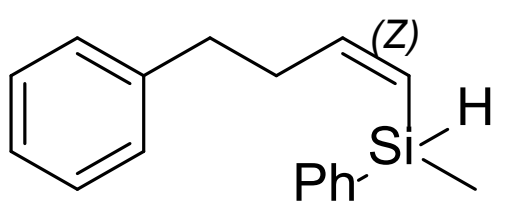

4-phenyl-1-butyne (70 µL, 0.5 mmol, 1 equiv.); methylphenylsilane (76 µL, 0.55 mmol, 1.1 equiv.); catalyst (2.2 mg, 1 mol%); eluent for thin pad of silica: petroleum ether; yield: 110 mg (87%) of a colorless oil. ^{1}H NMR (400 MHz, $CDCl_3$): δ = 7.61 – 7.52 (m, 2H), 7.43 – 7.33 (m, 3H), 7.31 – 7.25 (m, 2H), 7.23 – 7.17 (m, 1H), 7.15 – 7.10 (m, 2H), 6.57 (dt, *J* = 14.2, 7.3 Hz, 1H), 5.70 (ddt, *J* = 13.8, 5.1, 1.2 Hz, 1H), 4.79 – 4.70 (m, 1H), 2.76 – 2.64 (m, 2H), 2.59 – 2.51 (m, 2H), 0.40 (d, *J* = 3.9 Hz, 3H) ppm. ^{13}C{^{1}H} NMR (101 MHz, $CDCl_3$): δ = 150.8, 141.7, 136.4, 134.6, 129.4, 128.6, 128.4, 128.1, 126.0, 124.7, 35.9, 35.5, -4.1 ppm. ^{29}Si{^{1}H} NMR (79 MHz, $CDCl_3$): δ = -30.8 ppm.

[(1*Z*)-6-chloro-1-hexen-1-yl]methylphenylsilane (3k)

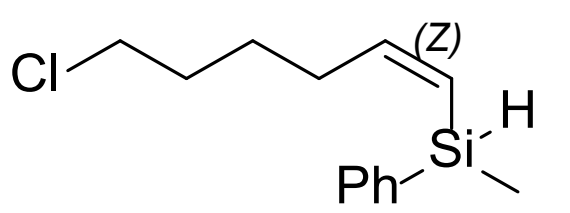

6-chloro-1-hexyne (61 µL, 0.5 mmol, 1 equiv.); methylphenylsilane (76 µL, 0.55 mmol, 1.1 equiv.); catalyst (2.2 mg, 1 mol%); eluent for thin pad of silica: petroleum ether; yield: 79 mg (66%) of a colorless oil. ^{1}H NMR (400 MHz, $CDCl_3$): δ = 7.61 – 7.51 (m, 2H), 7.44 – 7.31 (m, 3H), 6.49 (dt, *J* = 13.8, 7.4 Hz, 1H), 5.69 (ddt, *J* = 13.8, 5.1, 1.3 Hz, 1H), 4.79 – 4.70 (m, 1H), 3.48 (t, *J* = 6.7 Hz, 2H), 2.29 – 2.15 (m, 2H), 1.80 – 1.66 (m, 2H), 1.59 – 1.44 (m, 2H), 0.42 (d, *J* = 3.9 Hz, 3H) ppm. ^{13}C{1H} NMR (101 MHz, $CDCl_3$): δ = 151.1, 136.4, 134.5, 129.4, 128.1, 124.7, 45.0, 32.7, 32.1, 26.7, -4.0 ppm. ^{29}Si{^{1}H} NMR (79 MHz, $CDCl_3$): δ = -30.7 ppm.

[(1*Z*)-2-(1-cyclohexen-1-yl)ethen-1-yl)methylphenylsilane (3l)

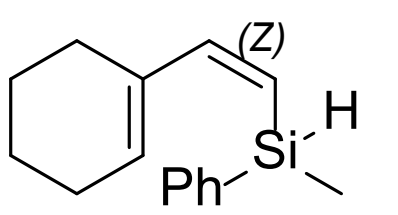

1-ethynyl-1-cyclohexene (59 µL, 0.5 mmol, 1 equiv.); methylphenylsilane (76 µL, 0.55 mmol, 1.1 equiv.); catalyst (2.2 mg, 1 mol%); conversion: 70% (according to GC-MS). This compound could not be isolated in pure form.

NMR Spectra

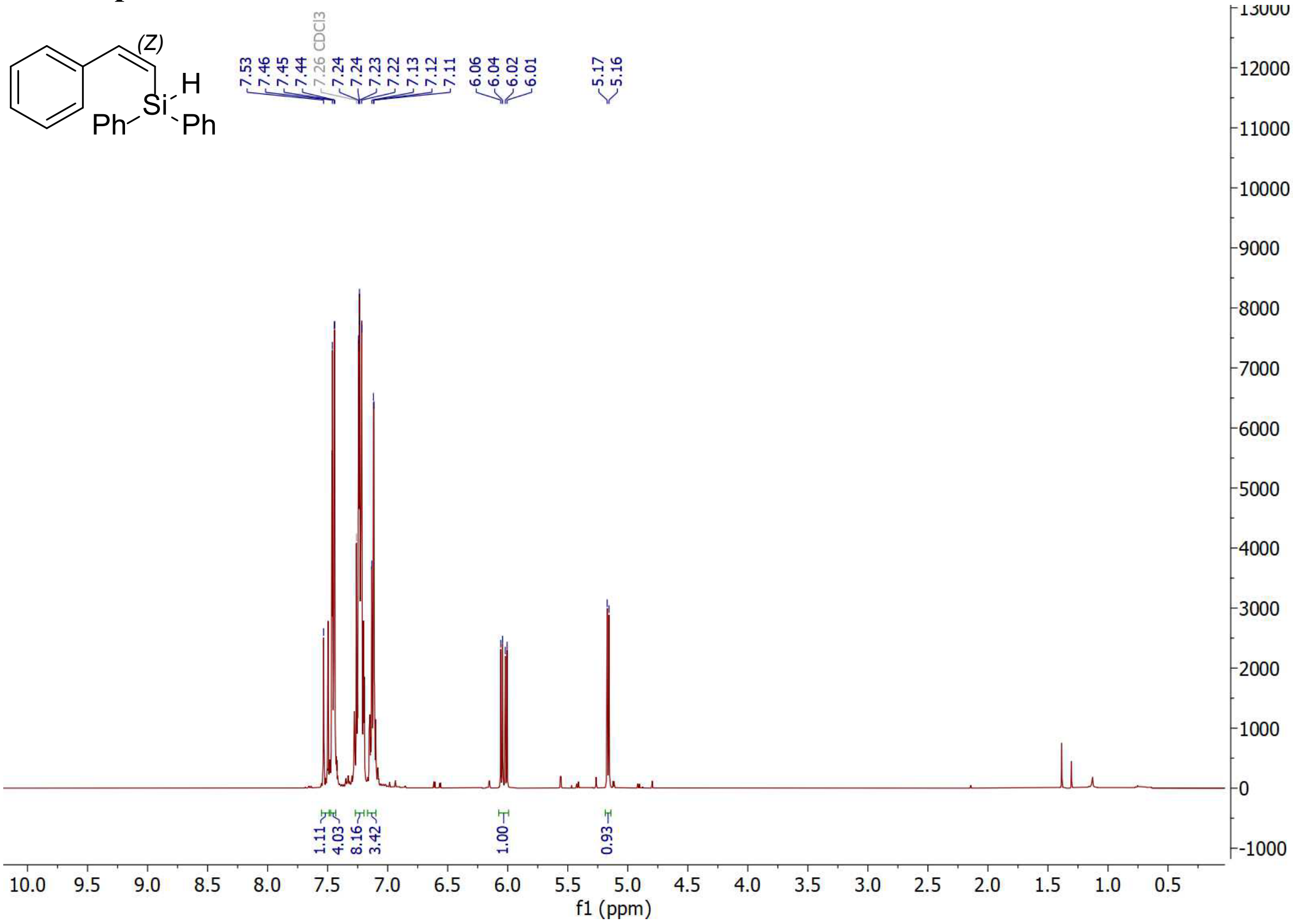

Figure S1. ^{1}H-NMR spectrum of **3a**

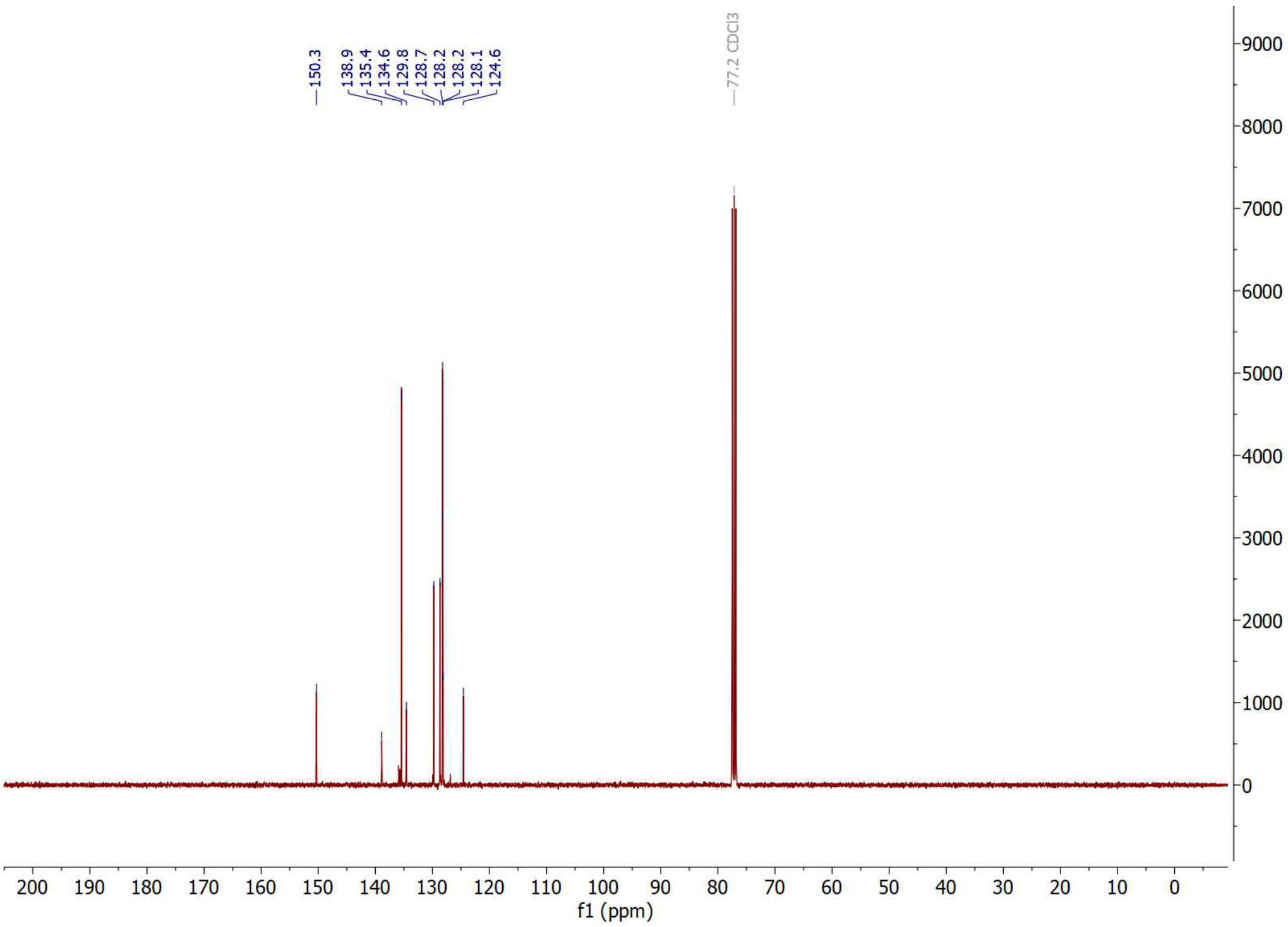

Figure s2. $^{13}C\{^{1}H\}$-NMR spectrum of **3a**

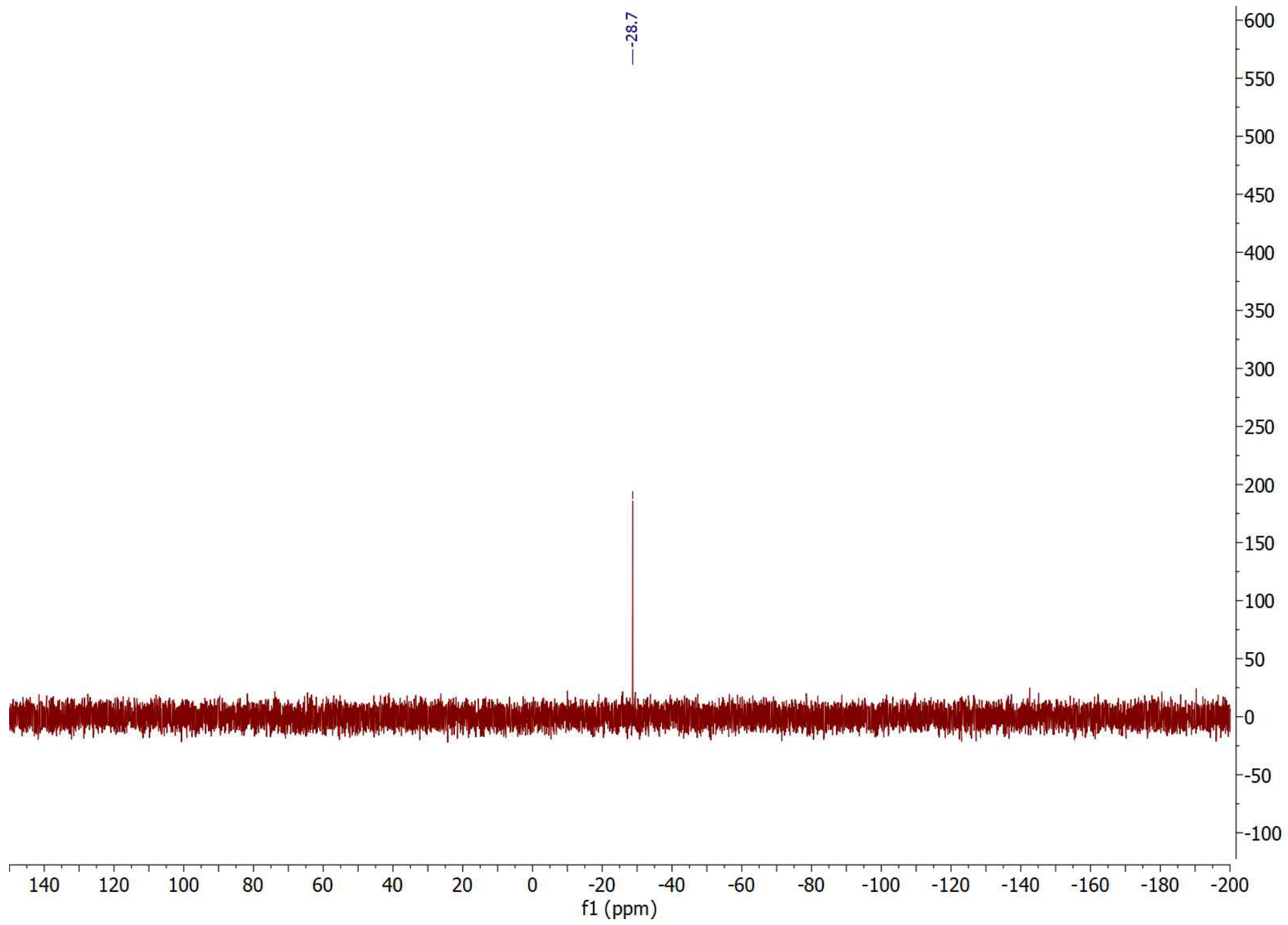

Figure S3. $^{29}Si\{^{1}H\}$-NMR spectrum of **3a**

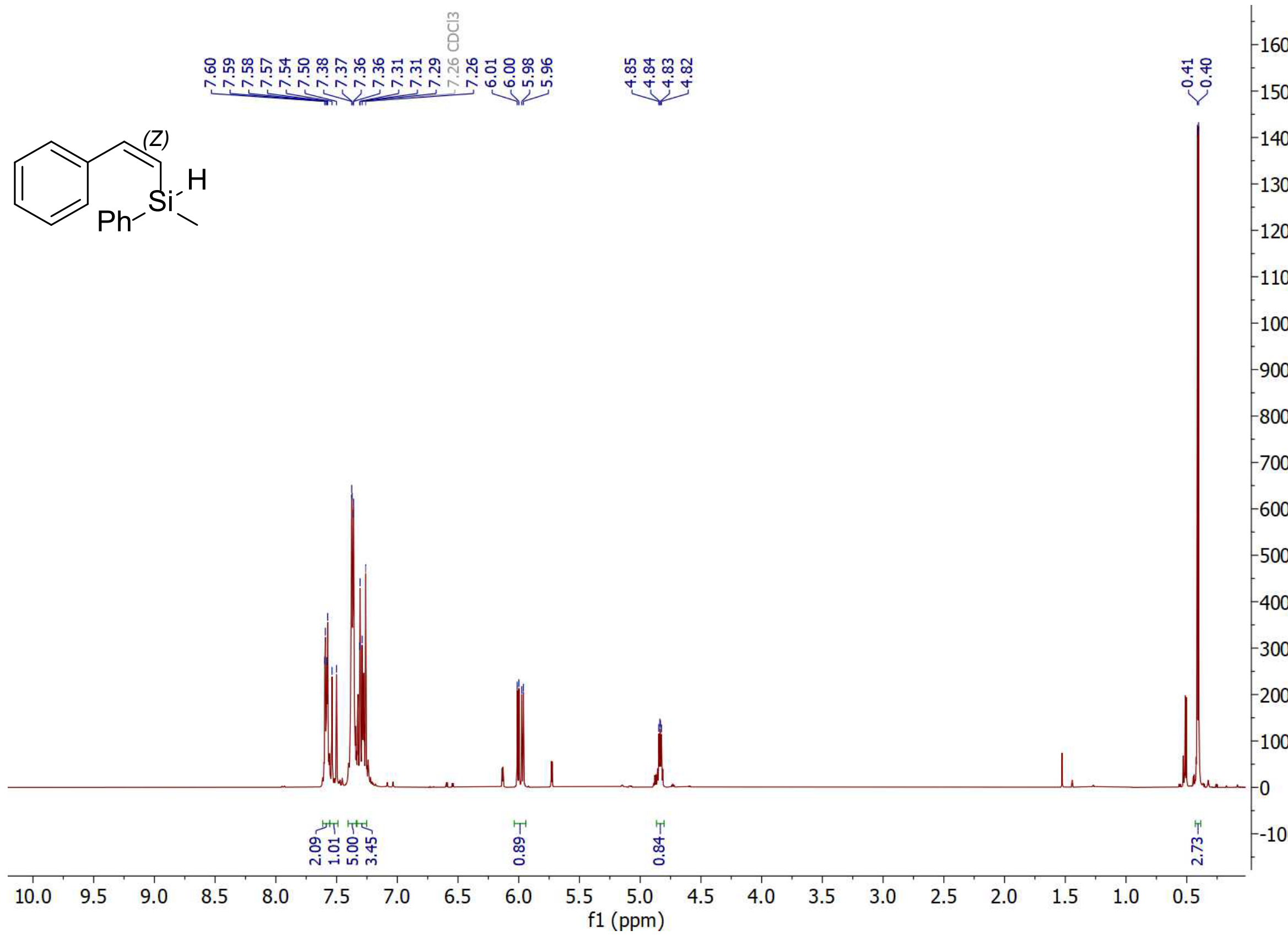

Figure S4. ^{1}H-NMR spectrum of **3b**

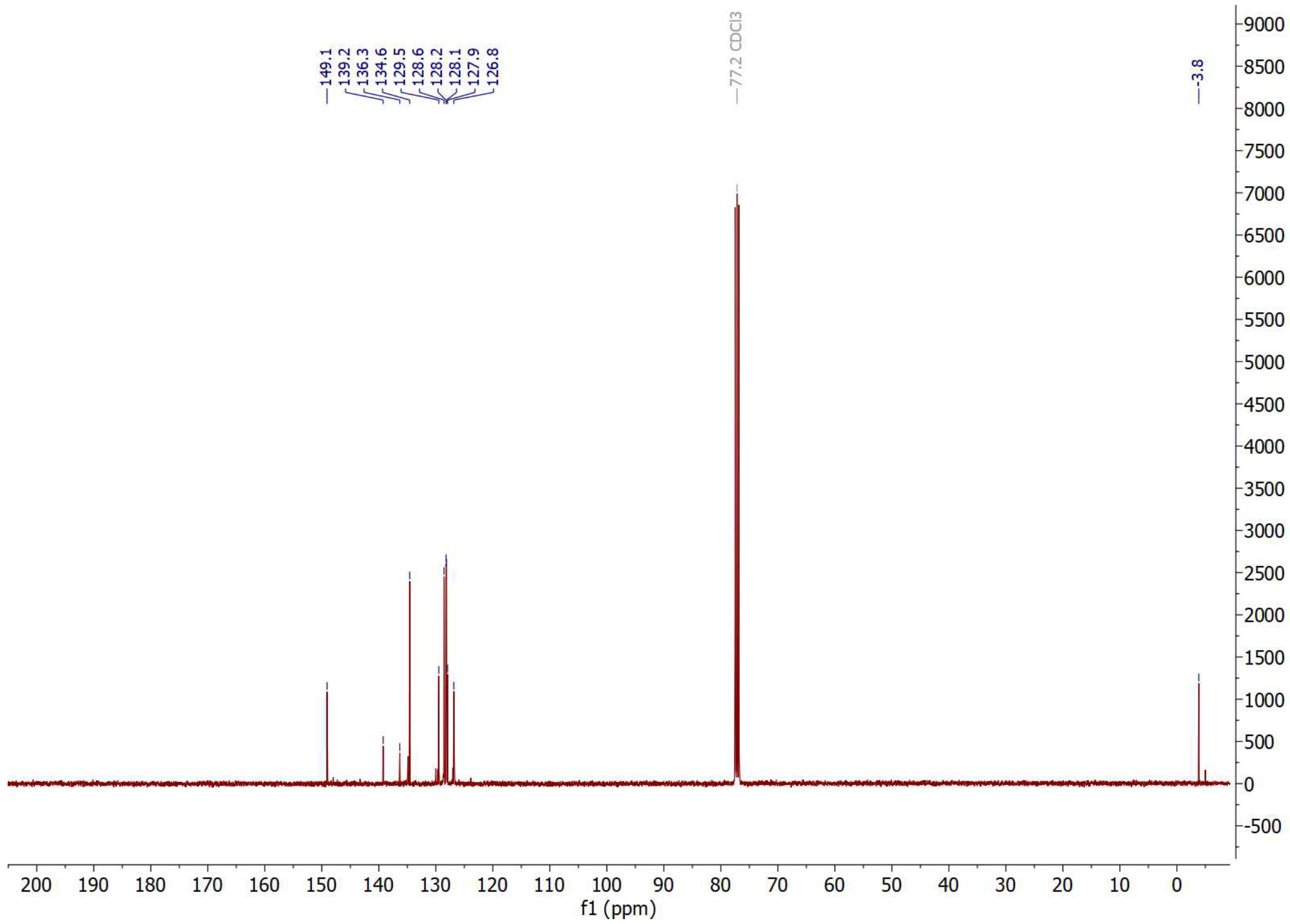

Figure S5. $^{13}C\{^{1}H\}$-NMR spectrum of **3b**

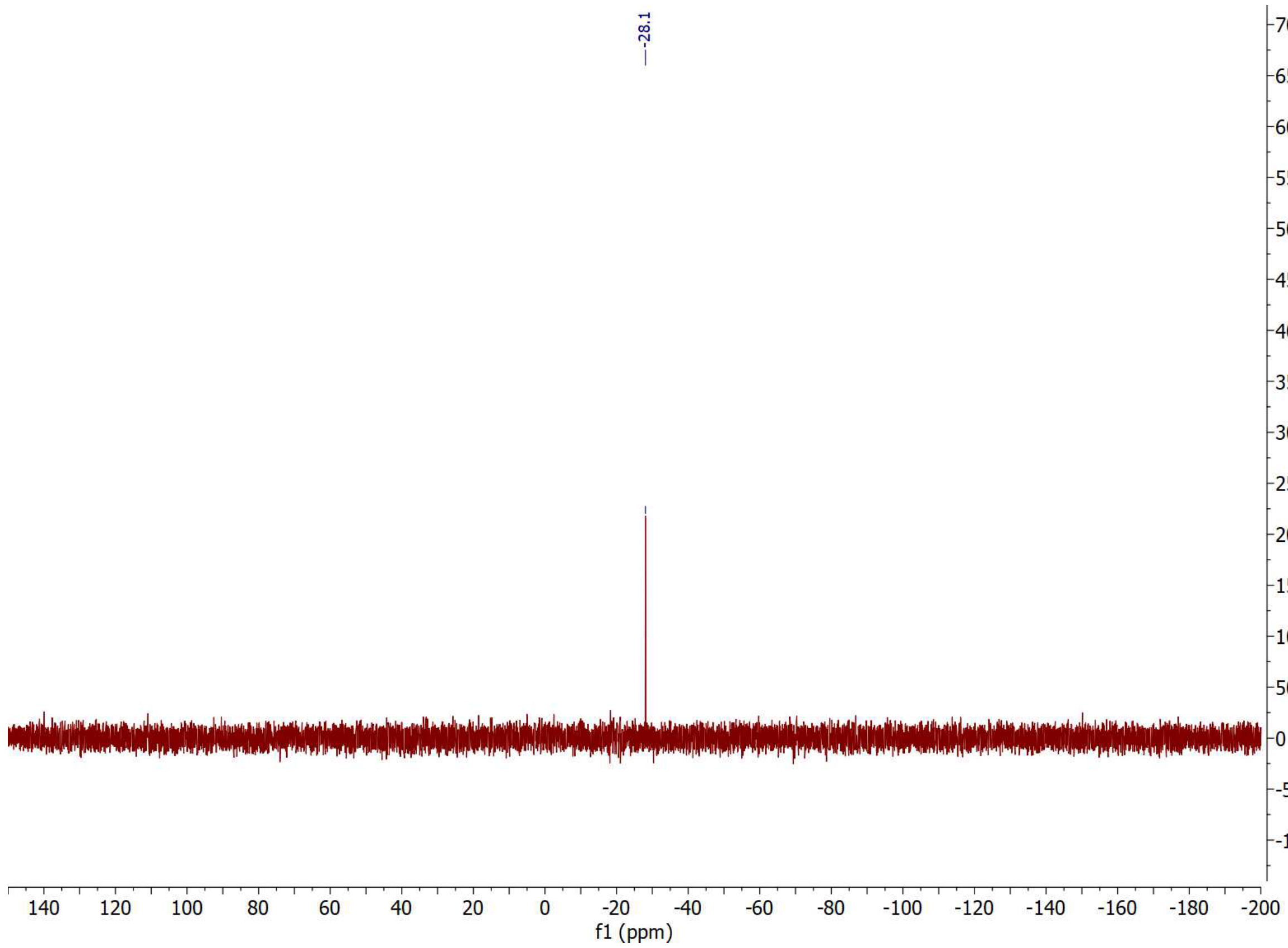

Figure S6. $^{29}Si\{^{1}H\}$-NMR spectrum of **3b**

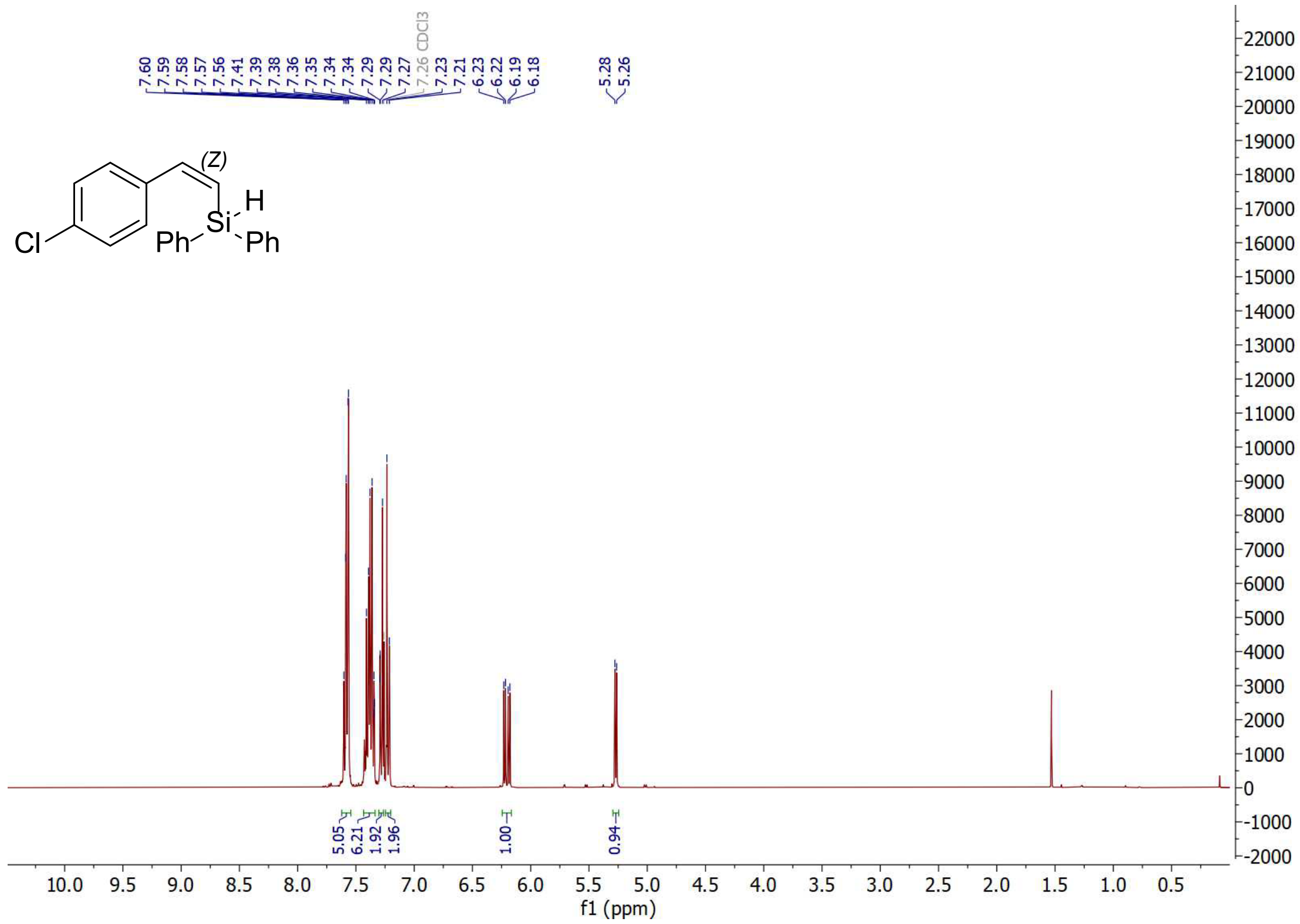

Figure S7. ^{1}H-NMR spectrum of **3d**

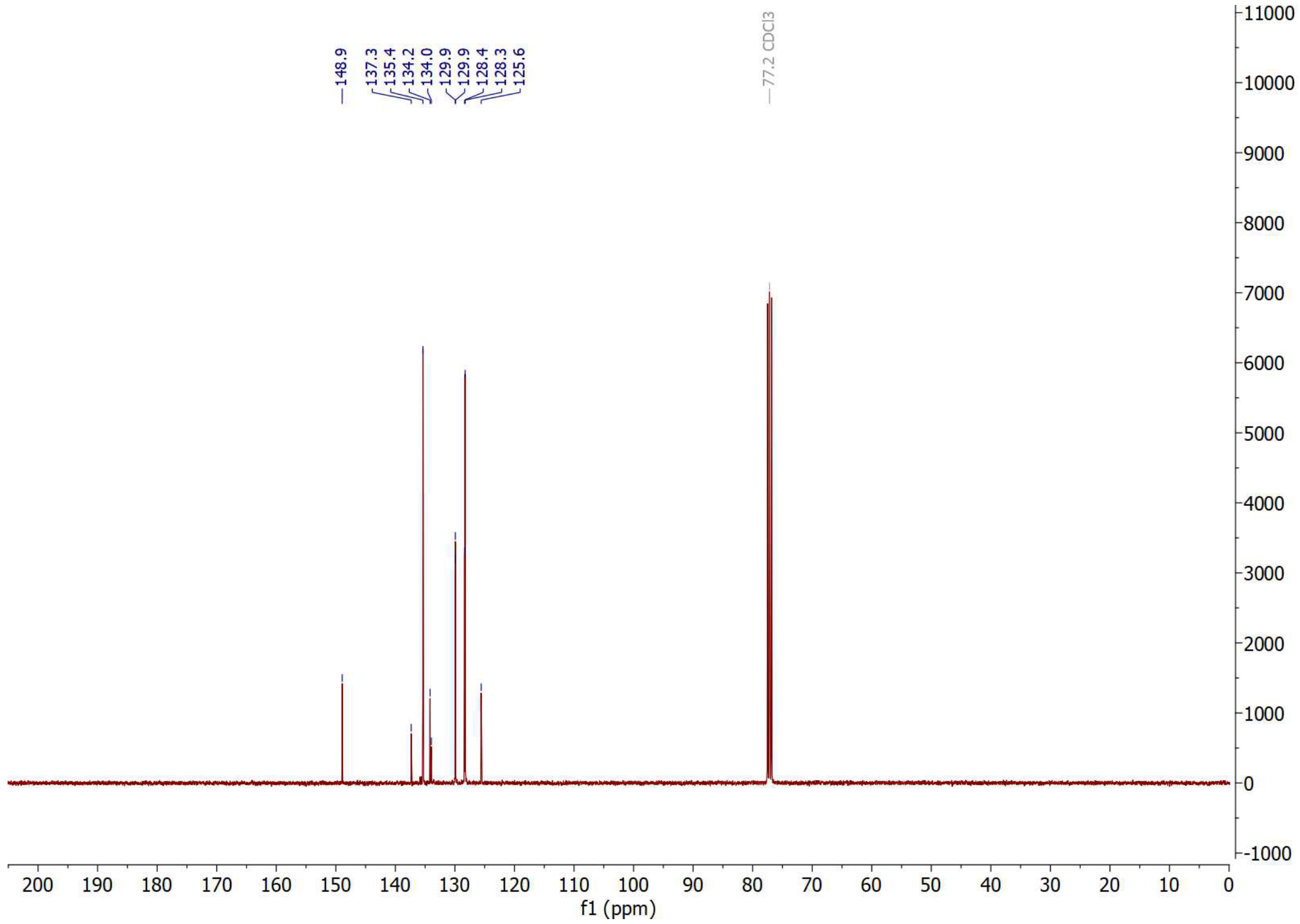

Figure S8. ^{13}C{^{1}H}-NMR spectrum of **3d**

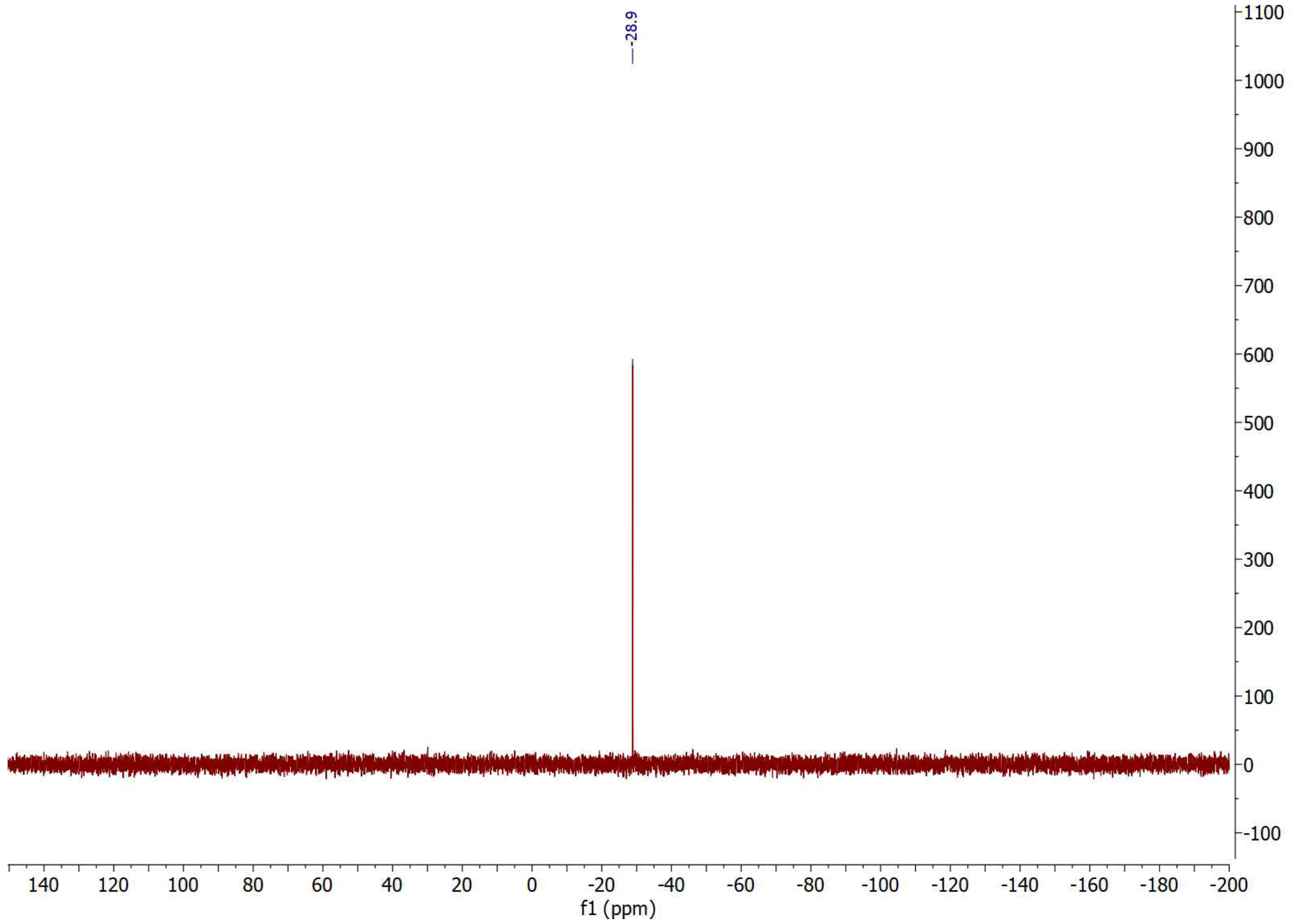

Figure S9. $^{29}Si\{^{1}H\}$-NMR spectrum of **3d**

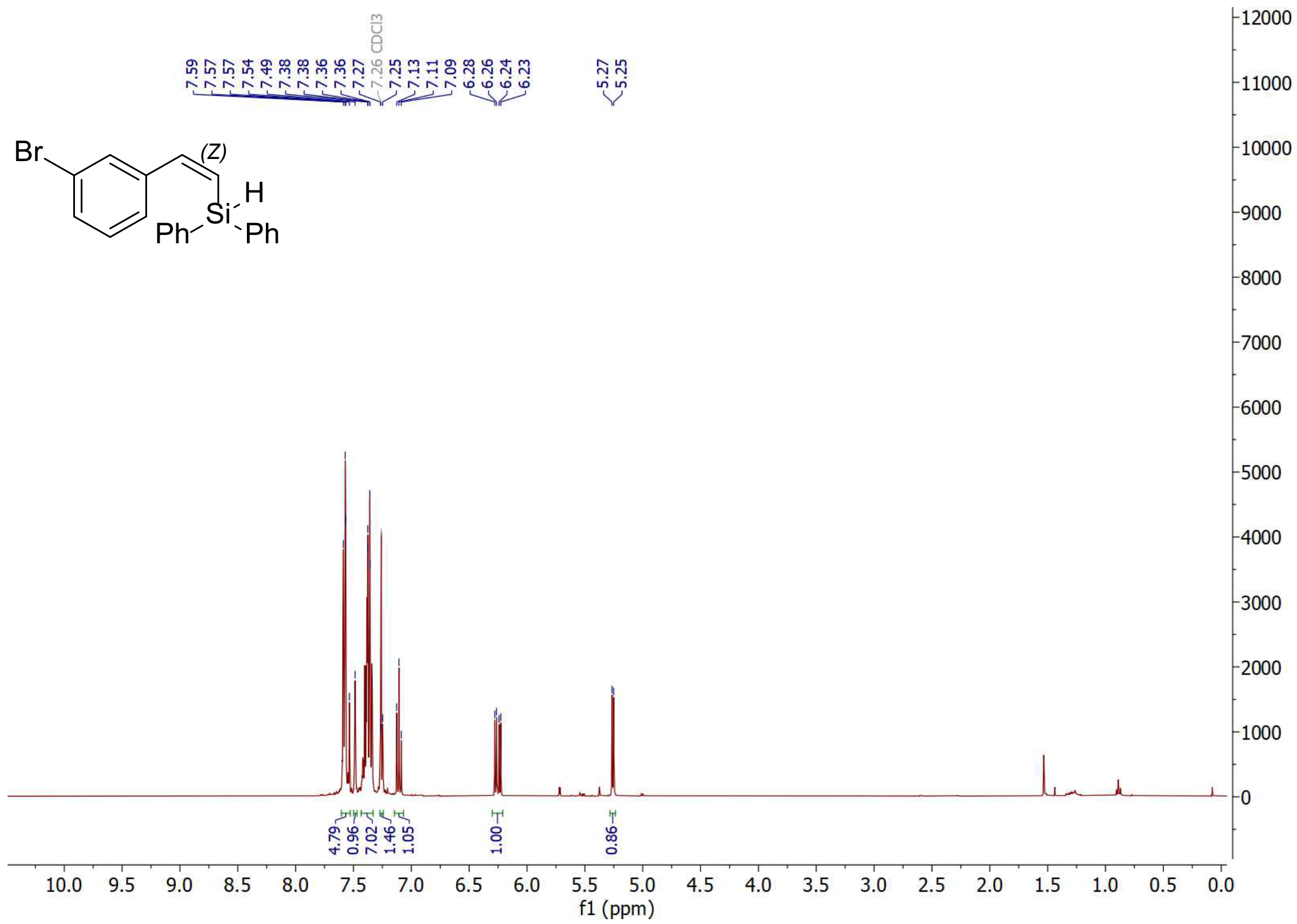

Figure S10. ^{1}H-NMR spectrum of **3e**

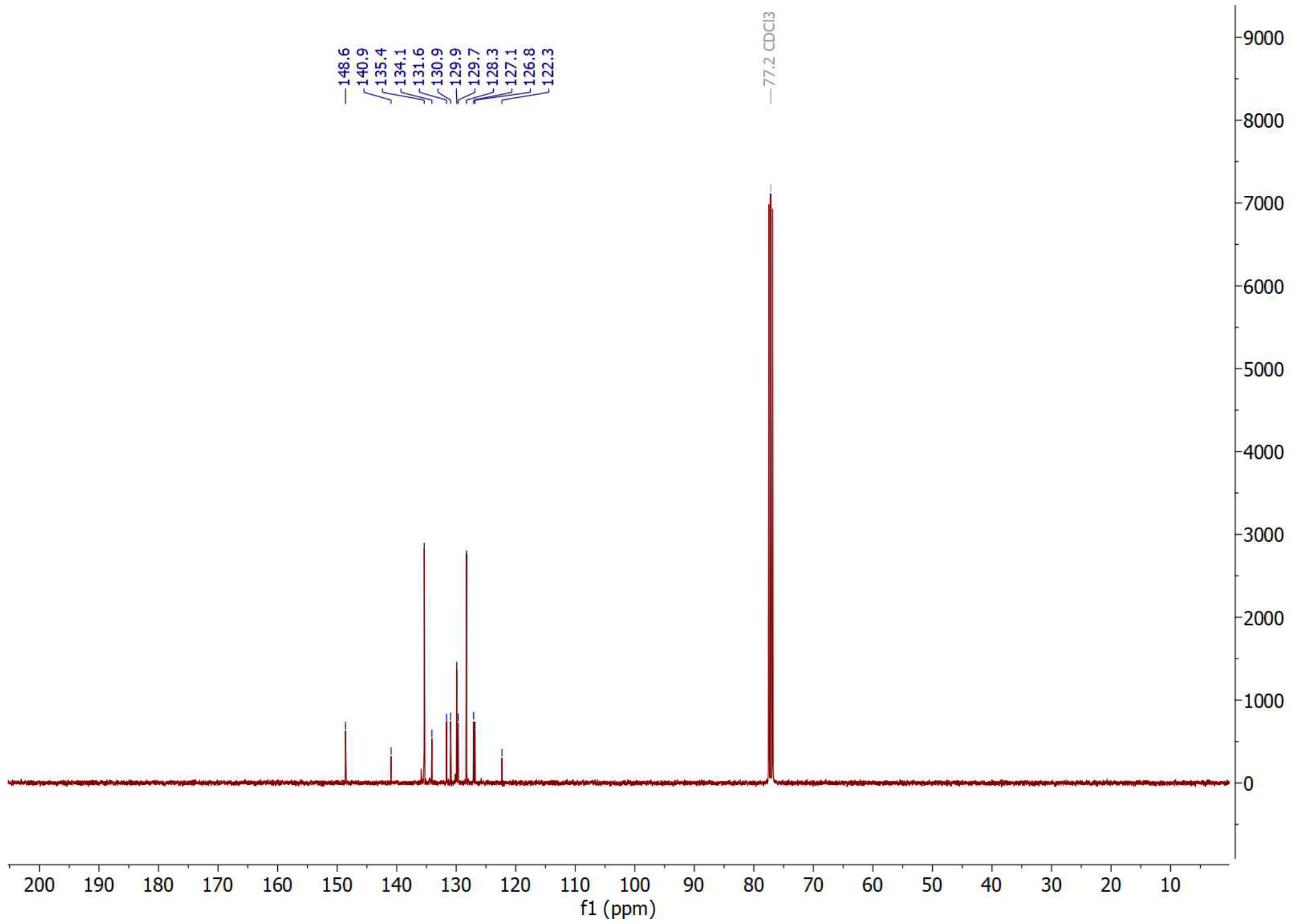

Figure S11. $^{13}C\{^{1}H\}$-NMR spectrum of **3e**

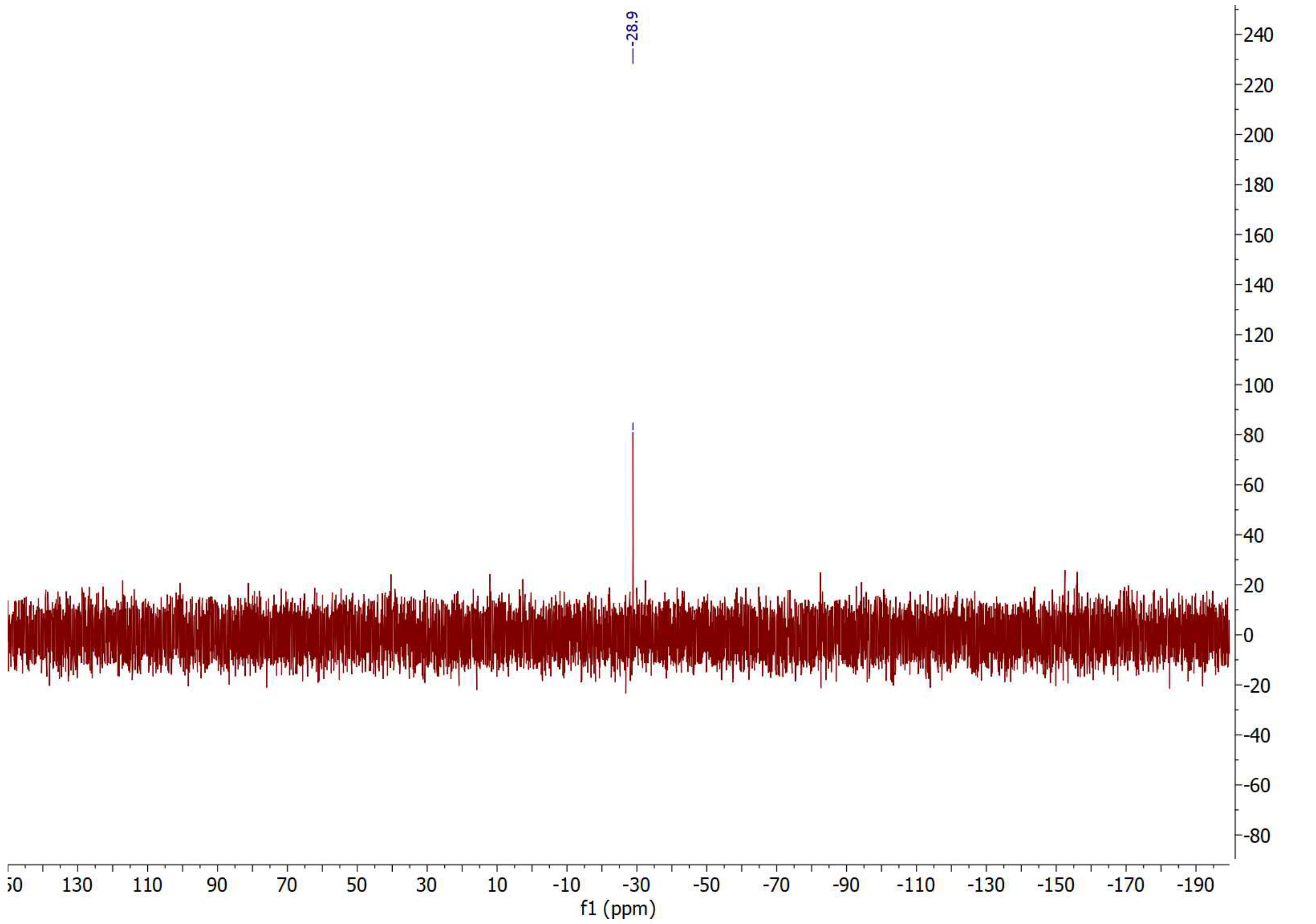

Figure S12. $^{29}Si\{^{1}H\}$-NMR spectrum of **3e**

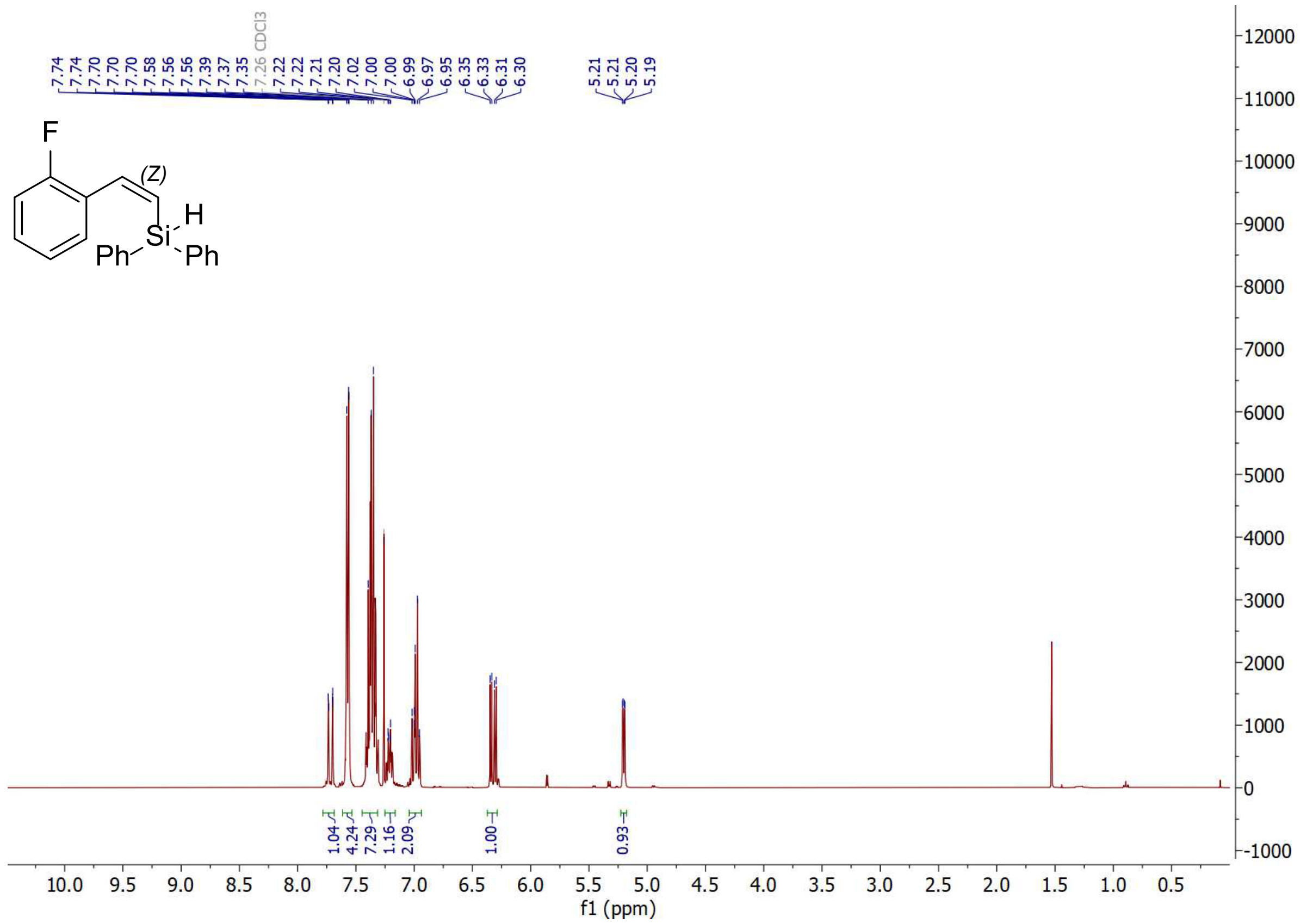

Figure S13. ^{1}H-NMR spectrum of **3f**

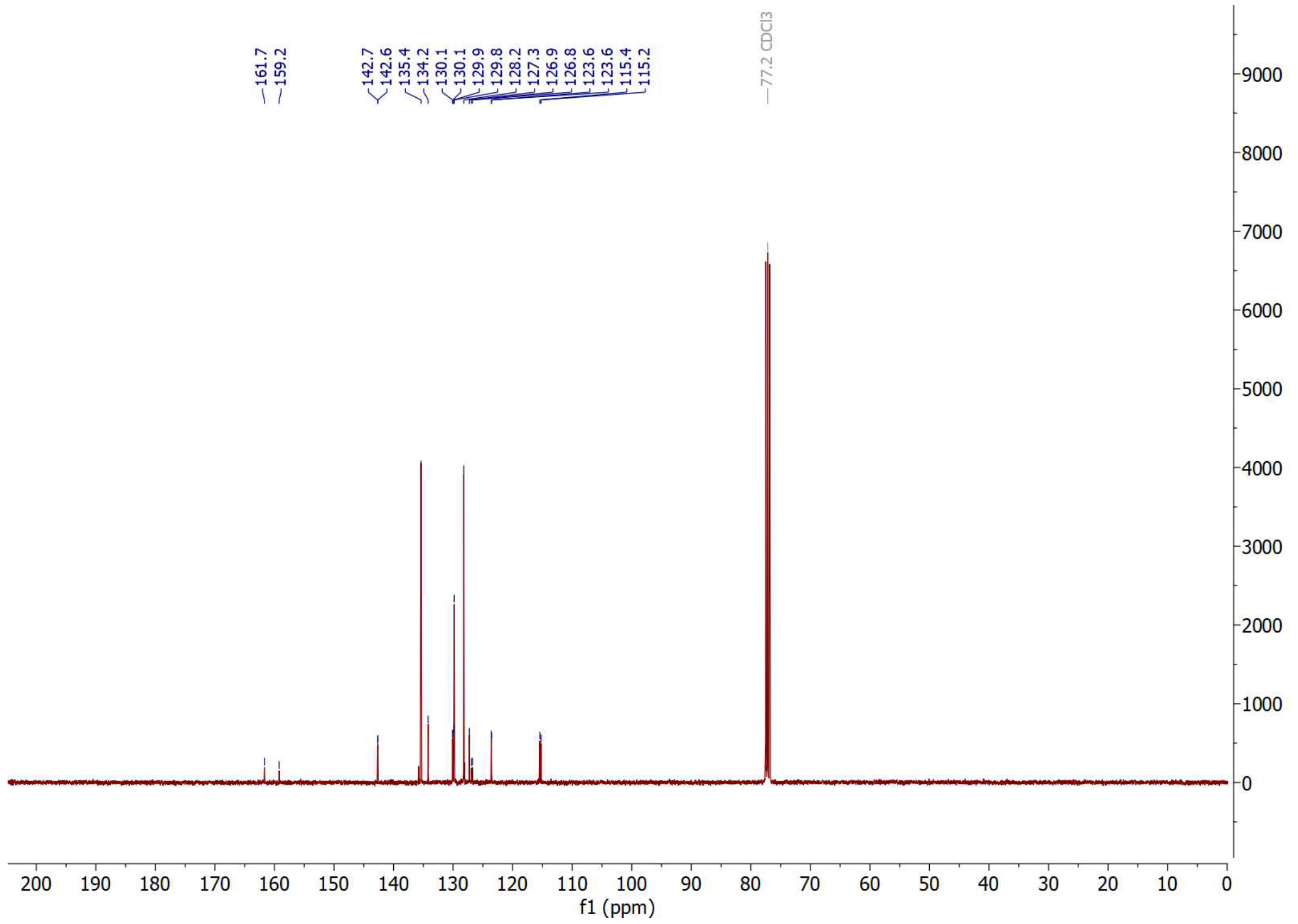

Figure S14. $^{13}C\{^{1}H\}$-NMR spectrum of **3f**

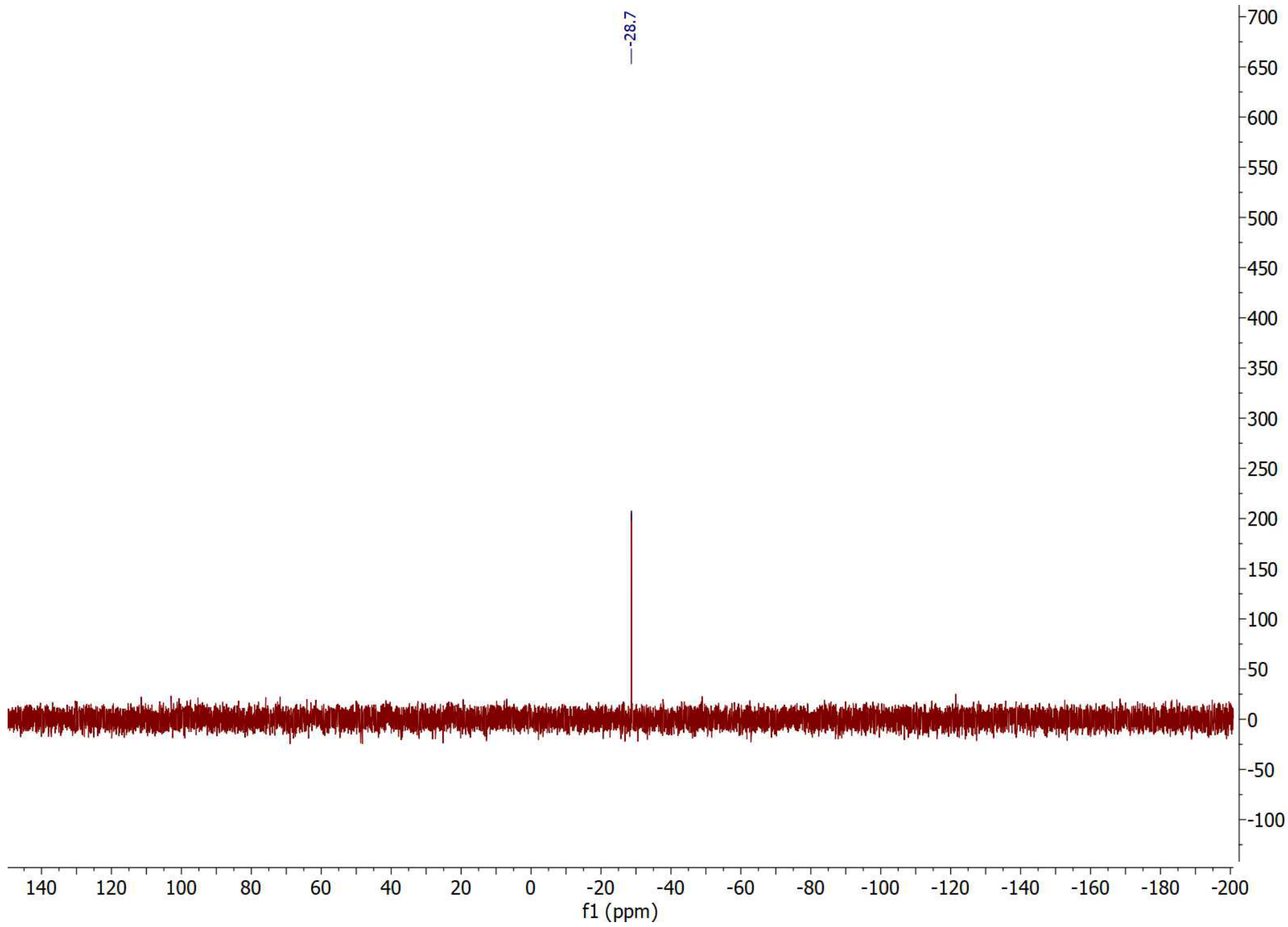

Figure S15. $^{29}Si\{^{1}H\}$-NMR spectrum of **3f**

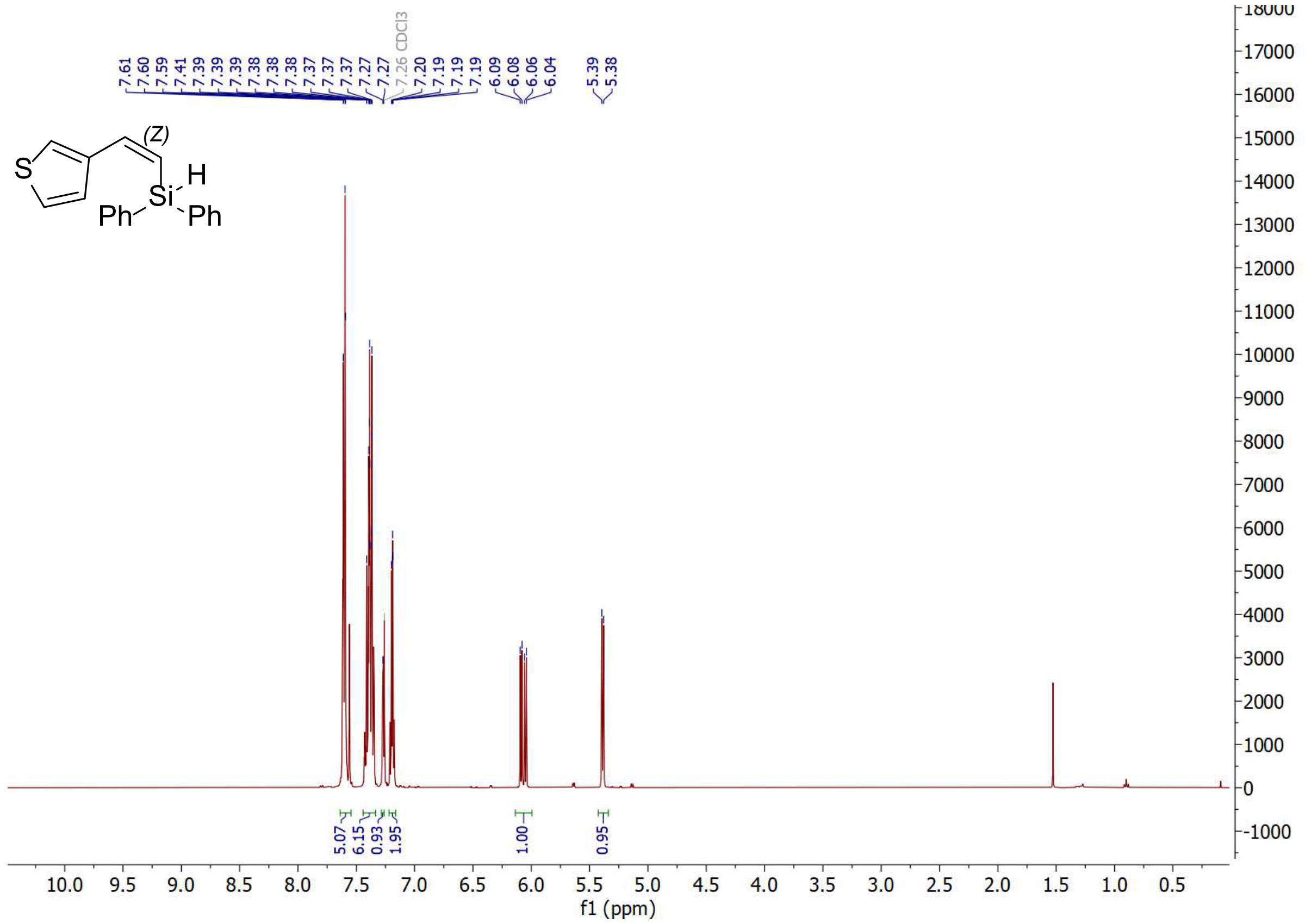

Figure S16. ^{1}H-NMR spectrum of **3g**

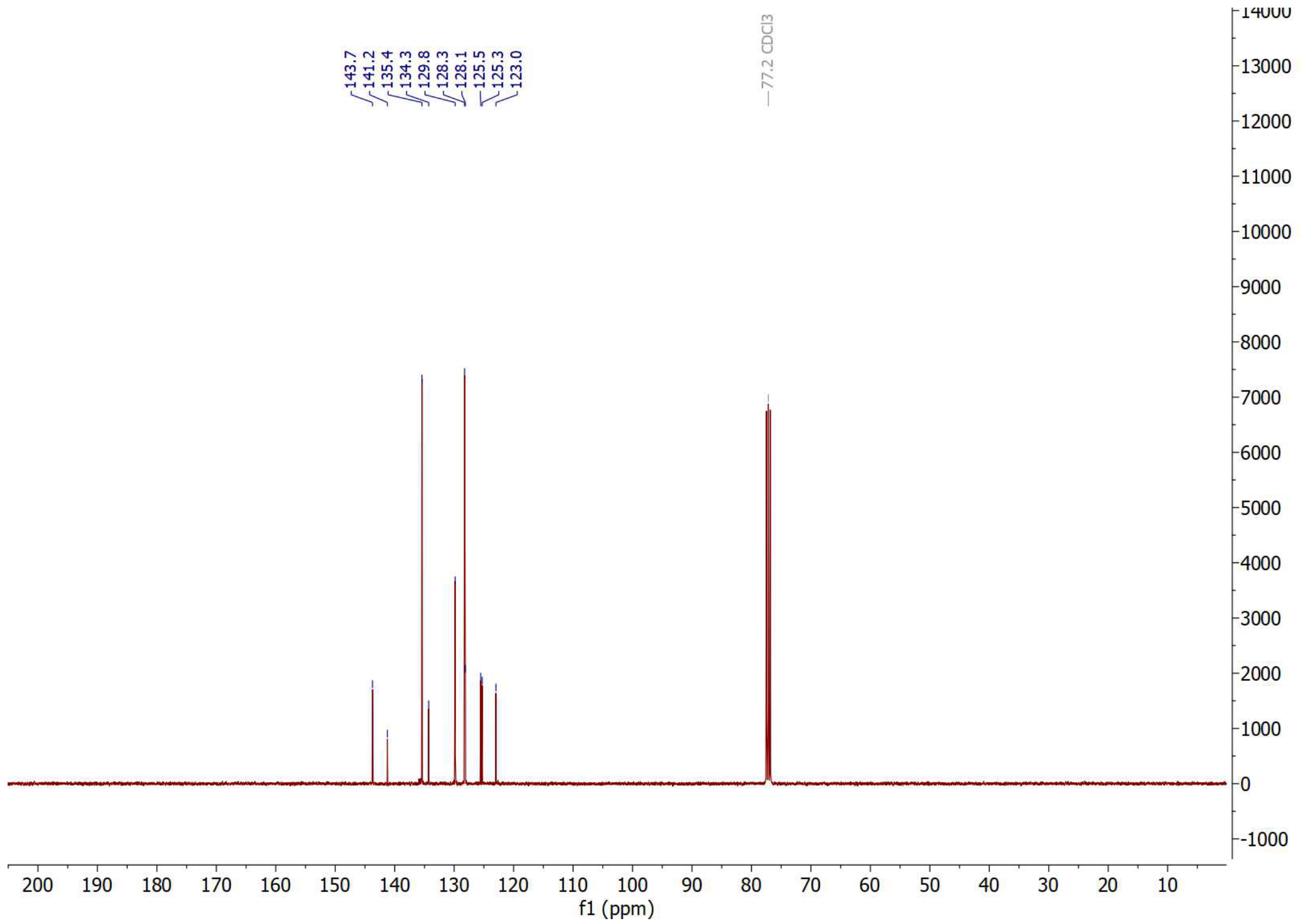

Figure S17. $^{13}C\{^{1}H\}$-NMR spectrum of **3g**

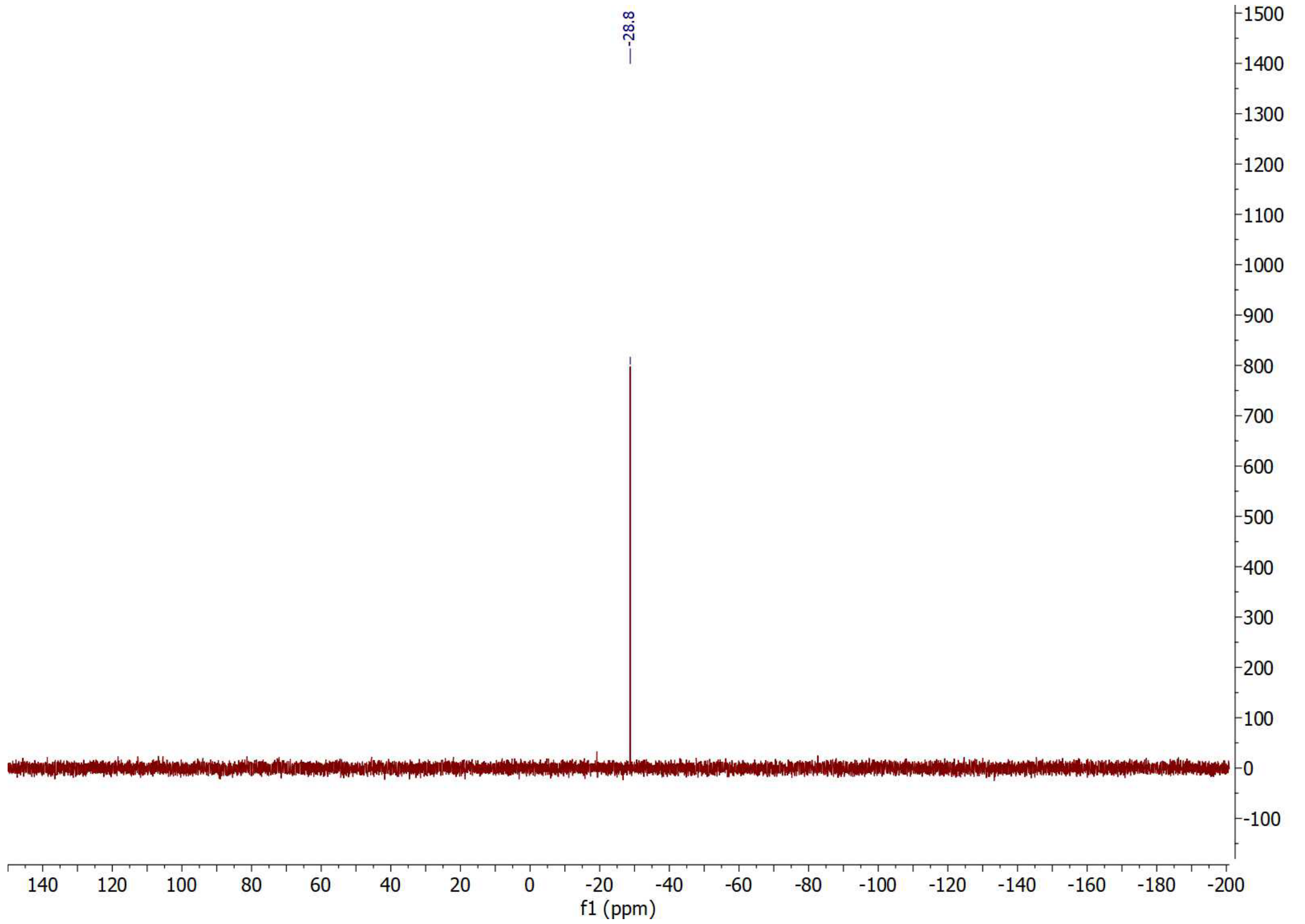

Figure S18. $^{29}Si\{^{1}H\}$-NMR spectrum of **3g**

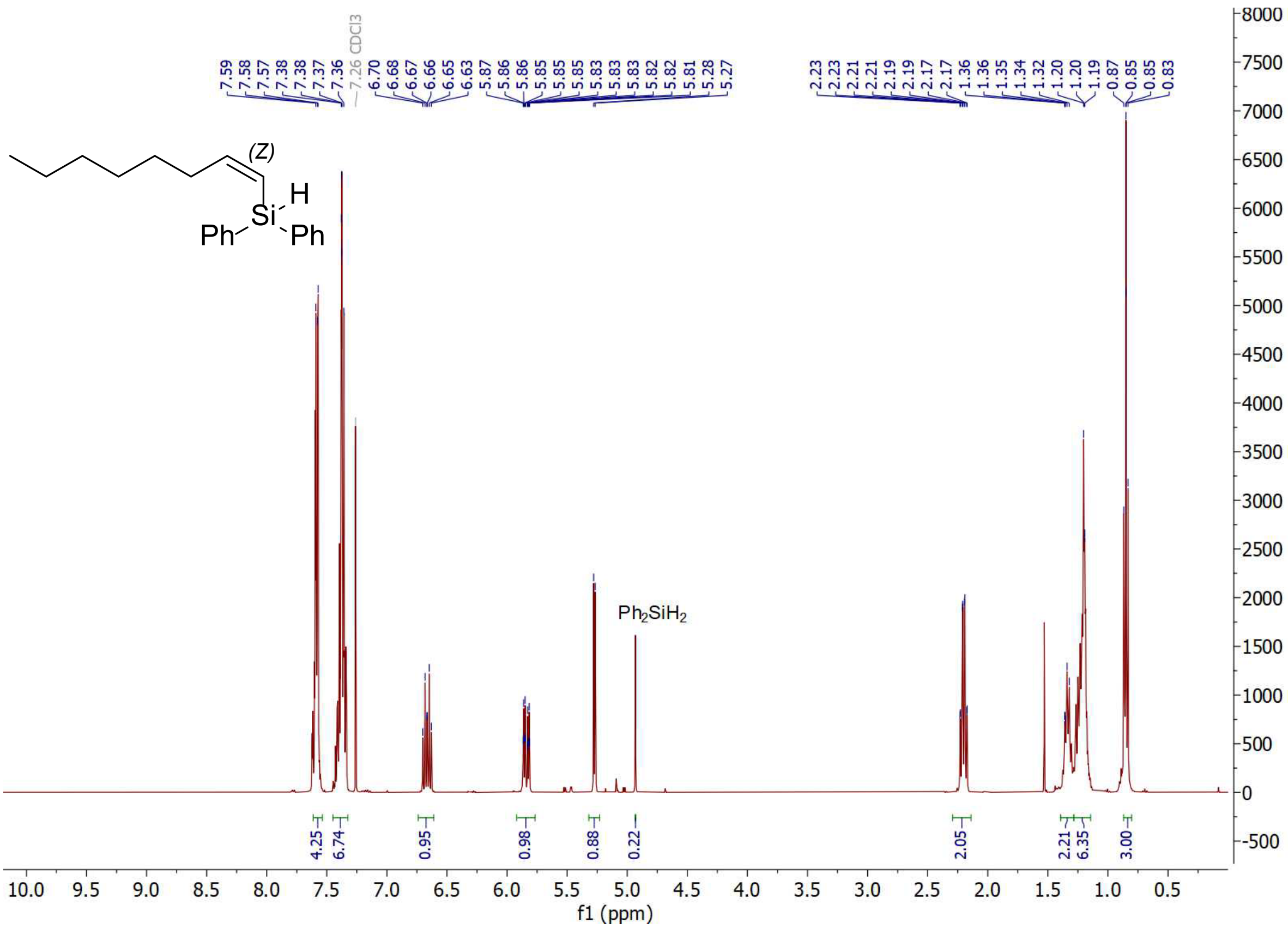

Figure S19. ^{1}H-NMR spectrum of **3h**

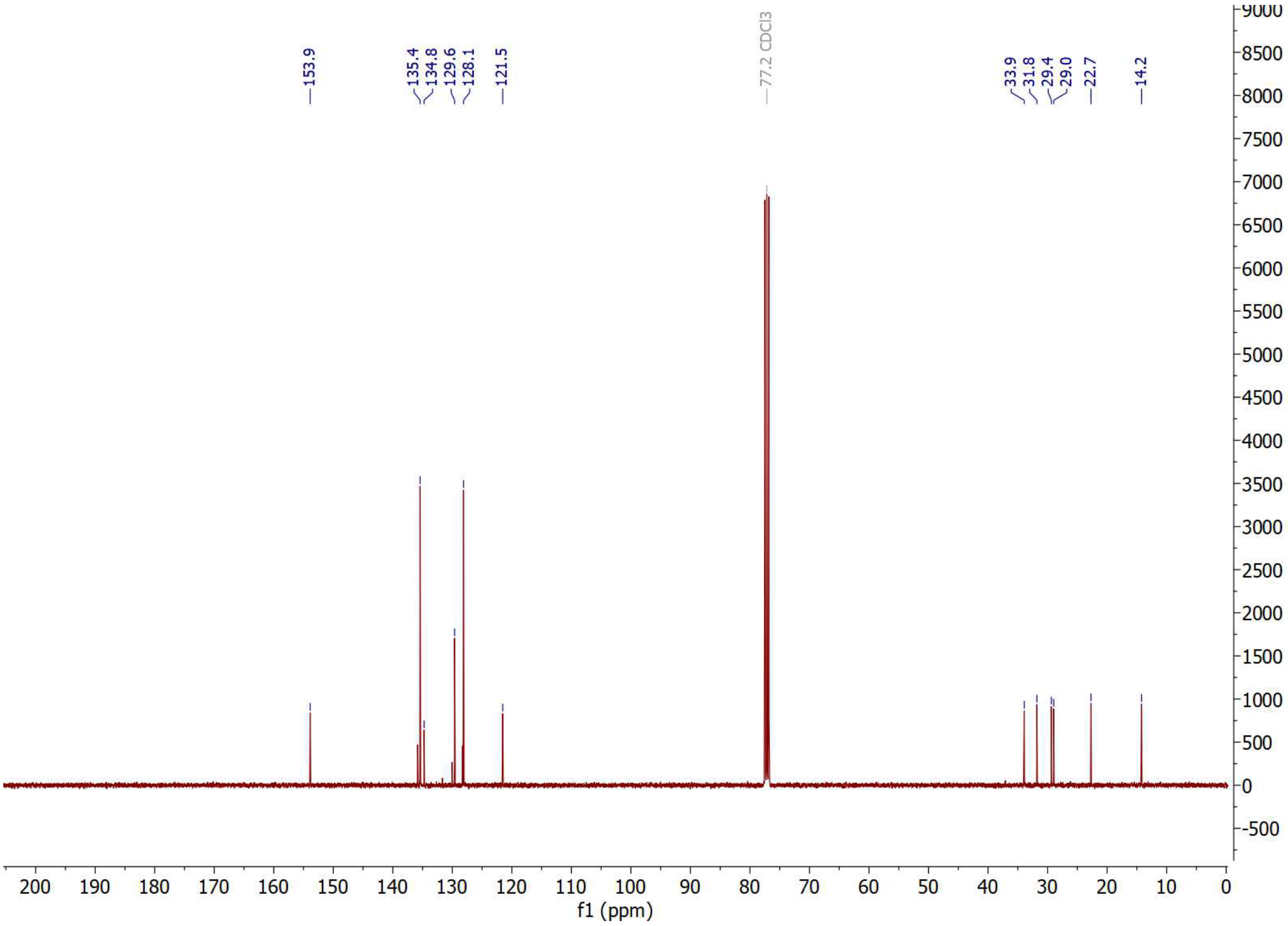

Figure S20. $^{13}C\{^{1}H\}$-NMR spectrum of **3h**

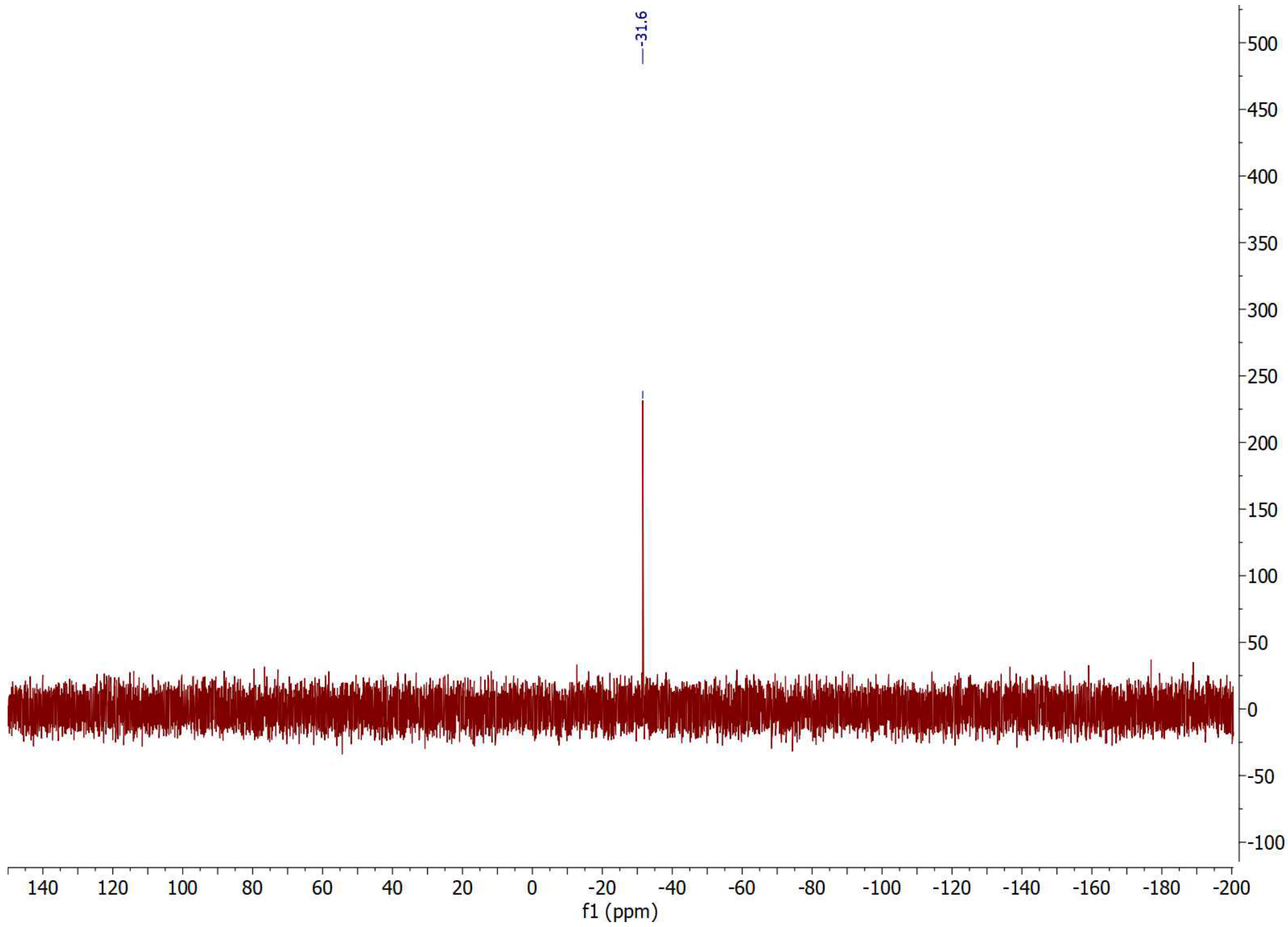

Figure S21. $^{29}Si\{^{1}H\}$-NMR spectrum of **3h**

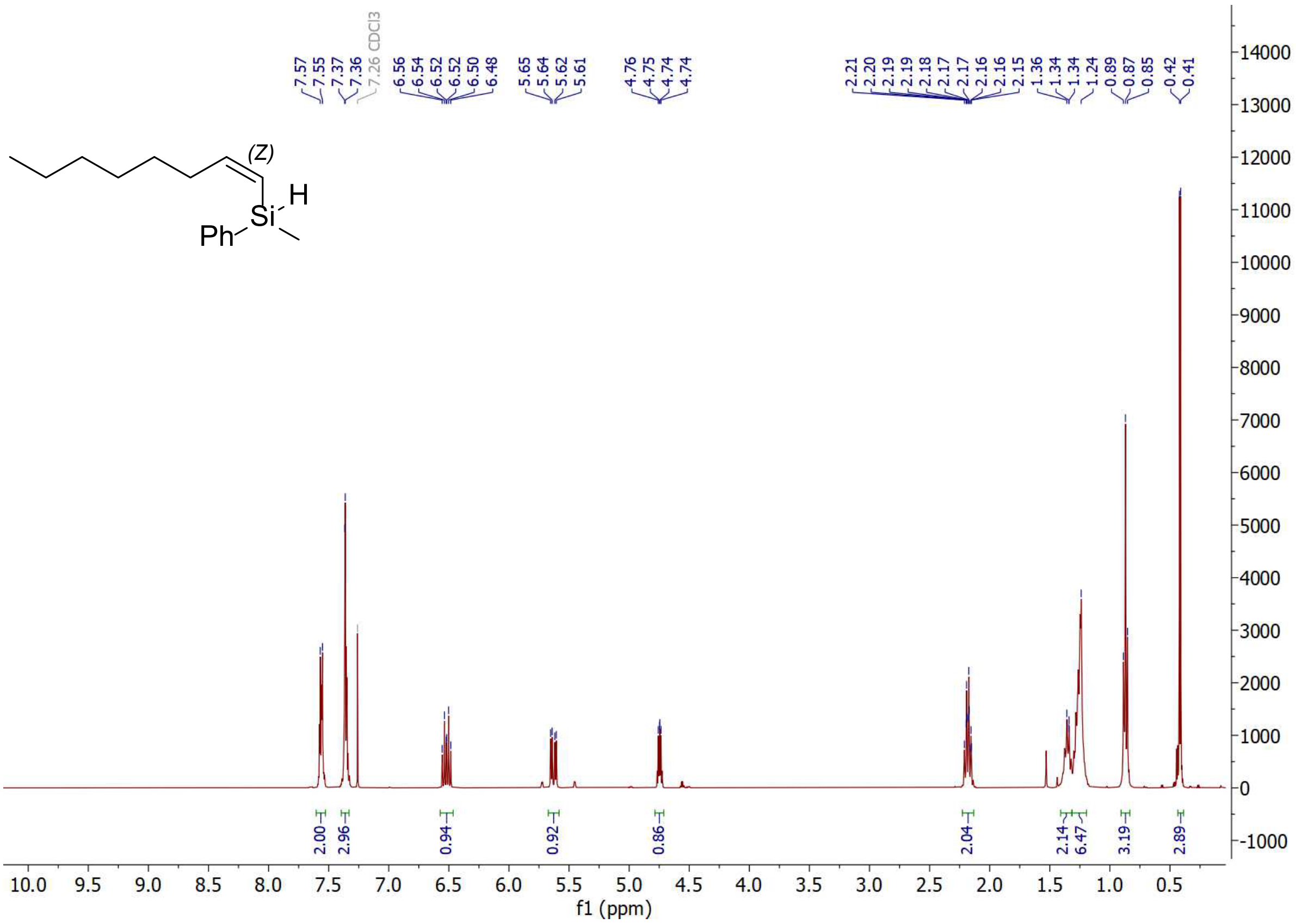

Figure S22. ^{1}H-NMR spectrum of **3i**

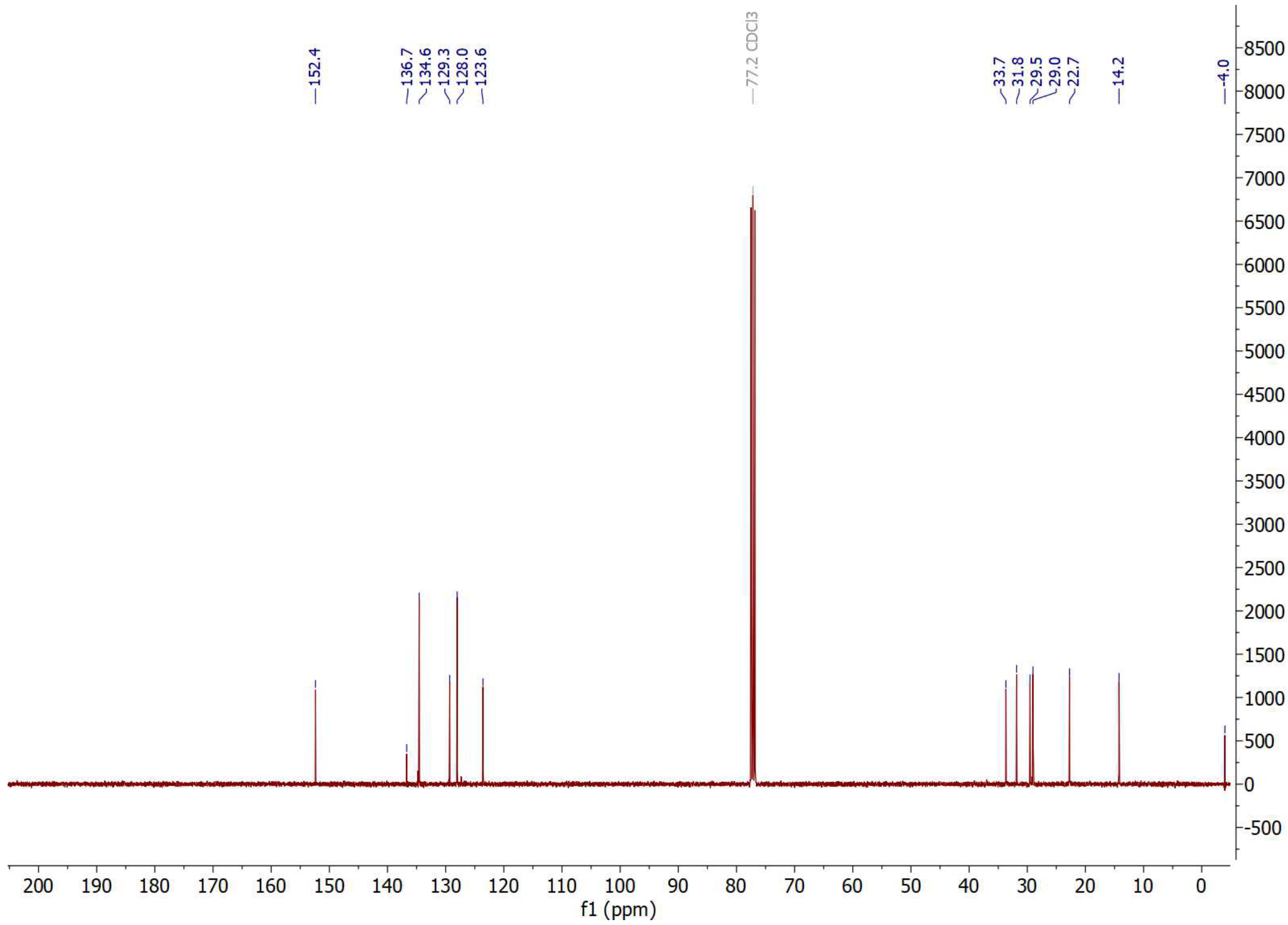

Figure S23. $^{13}C\{^1H\}$-NMR spectrum of **3i**

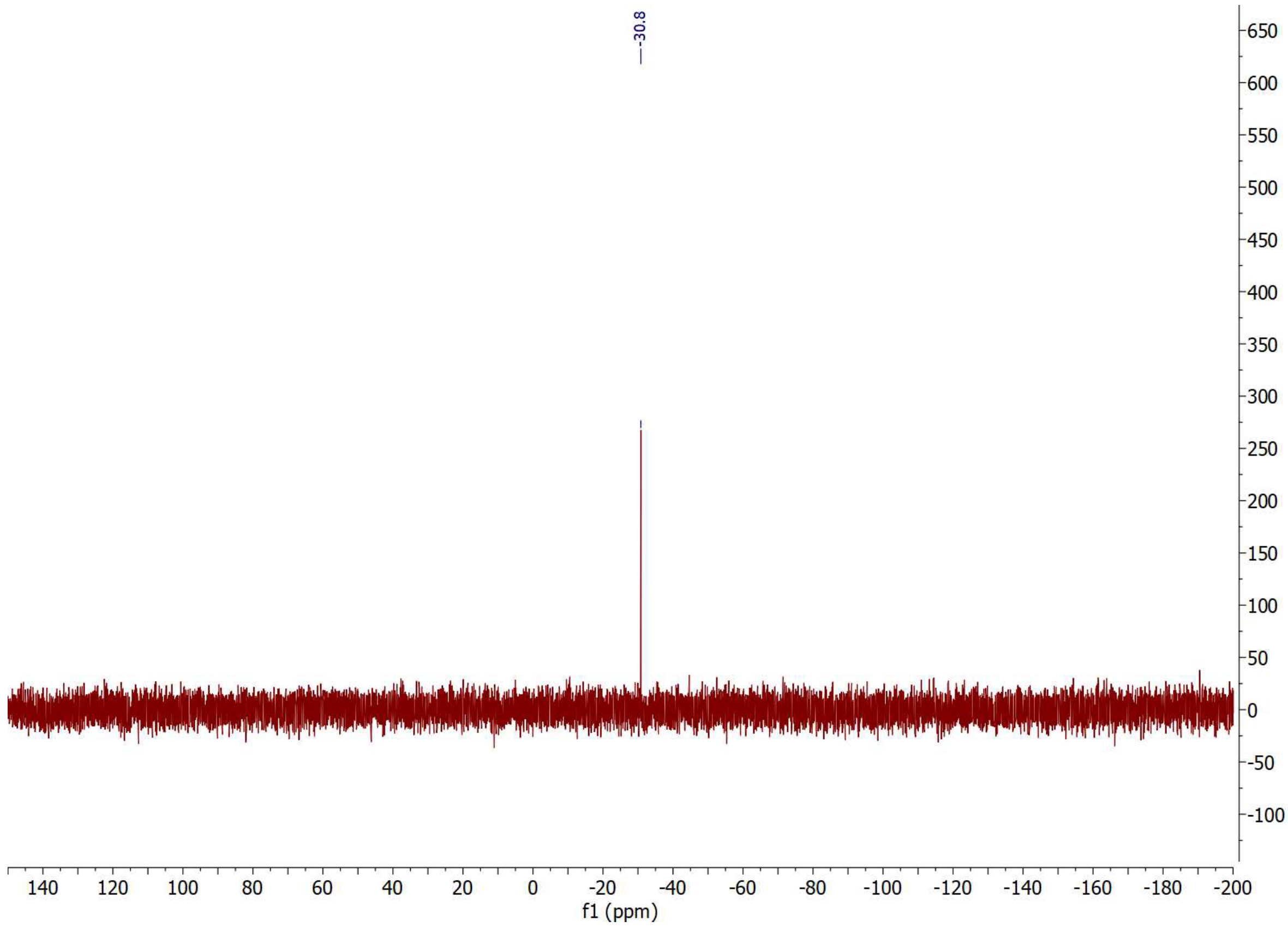

Figure S24. $^{29}Si\{^1H\}$-NMR spectrum of **3i**

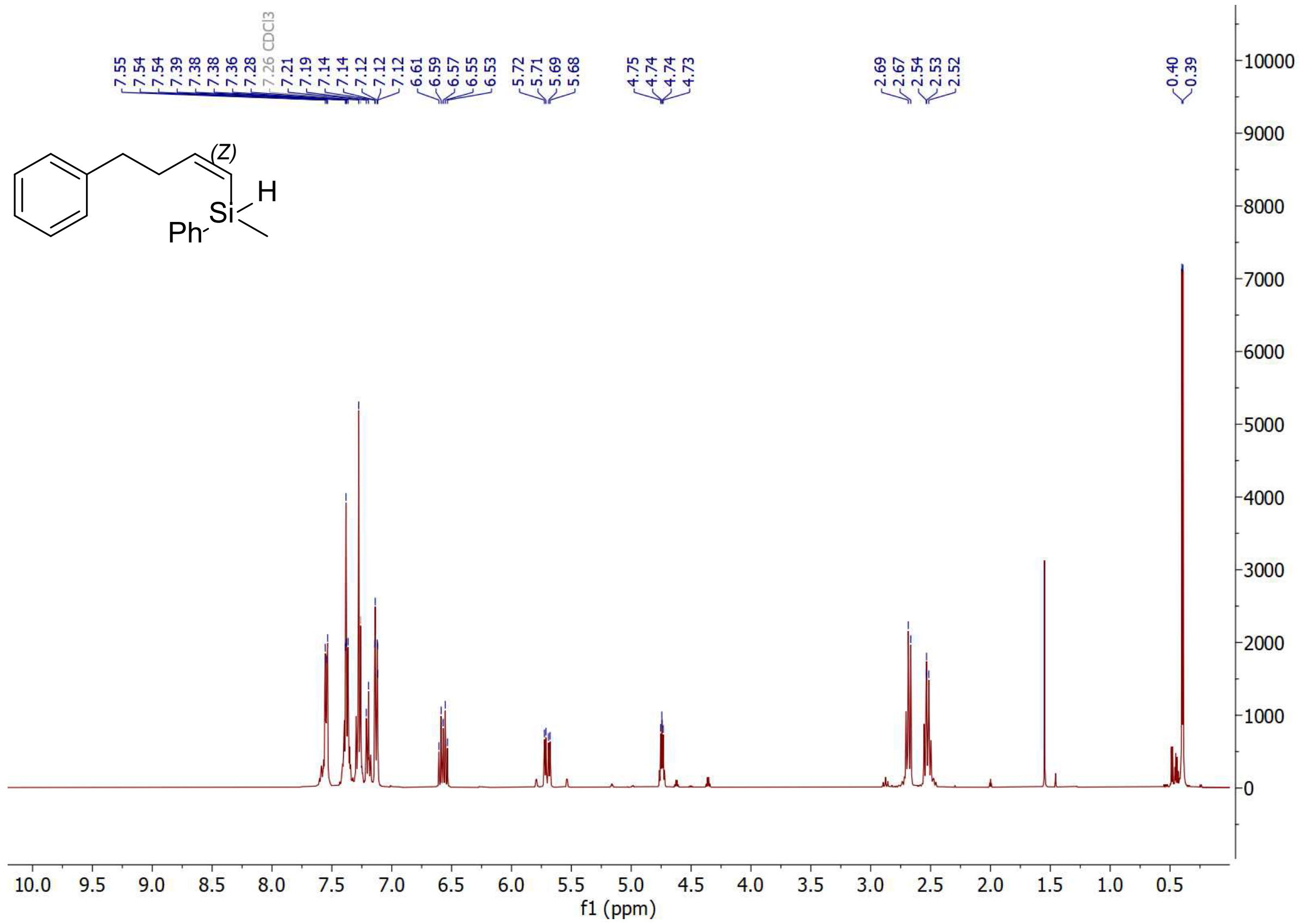

Figure S25. ^{1}H-NMR spectrum of **3j**

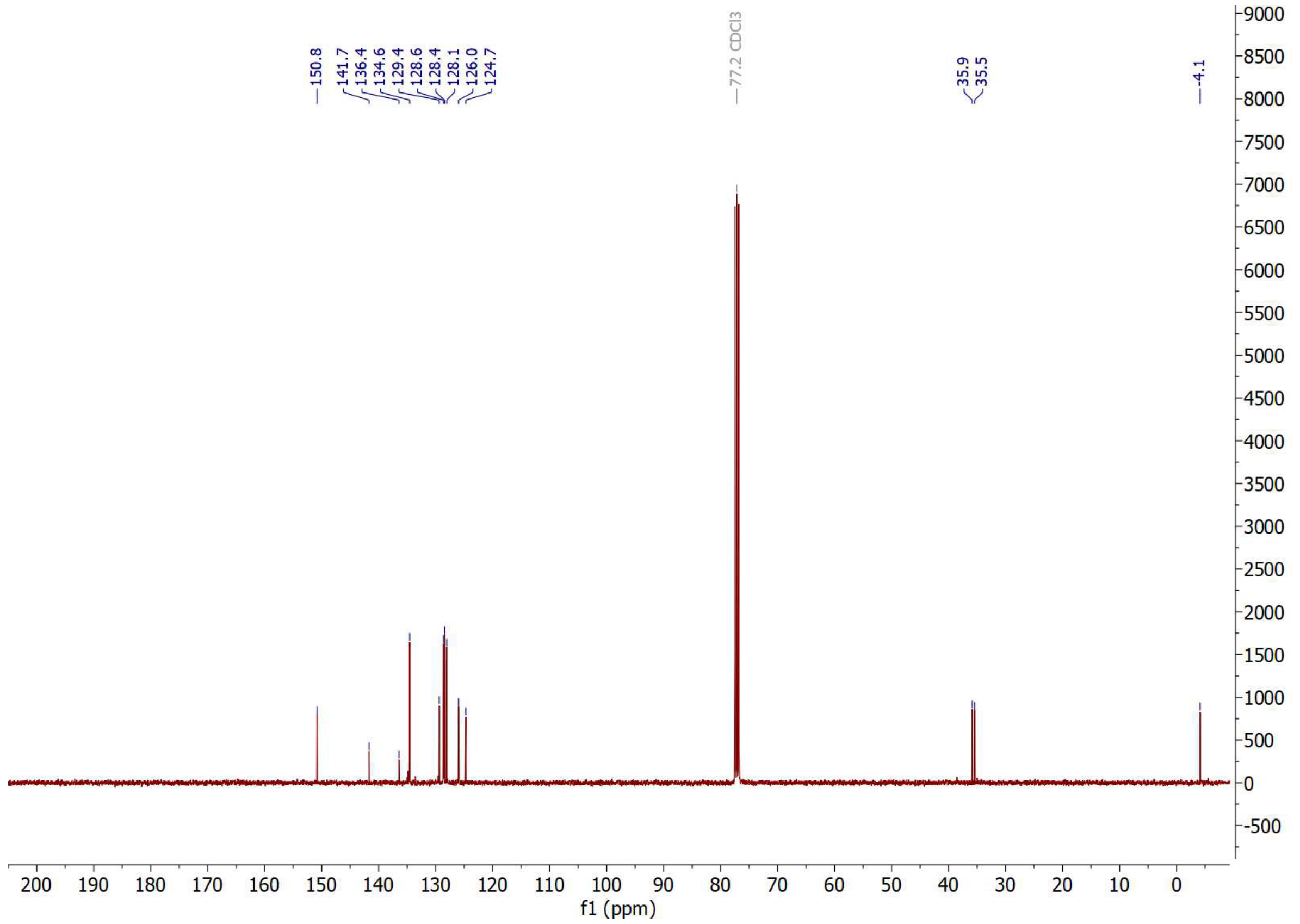

Figure S26. $^{13}C\{^{1}H\}$-NMR spectrum of **3j**

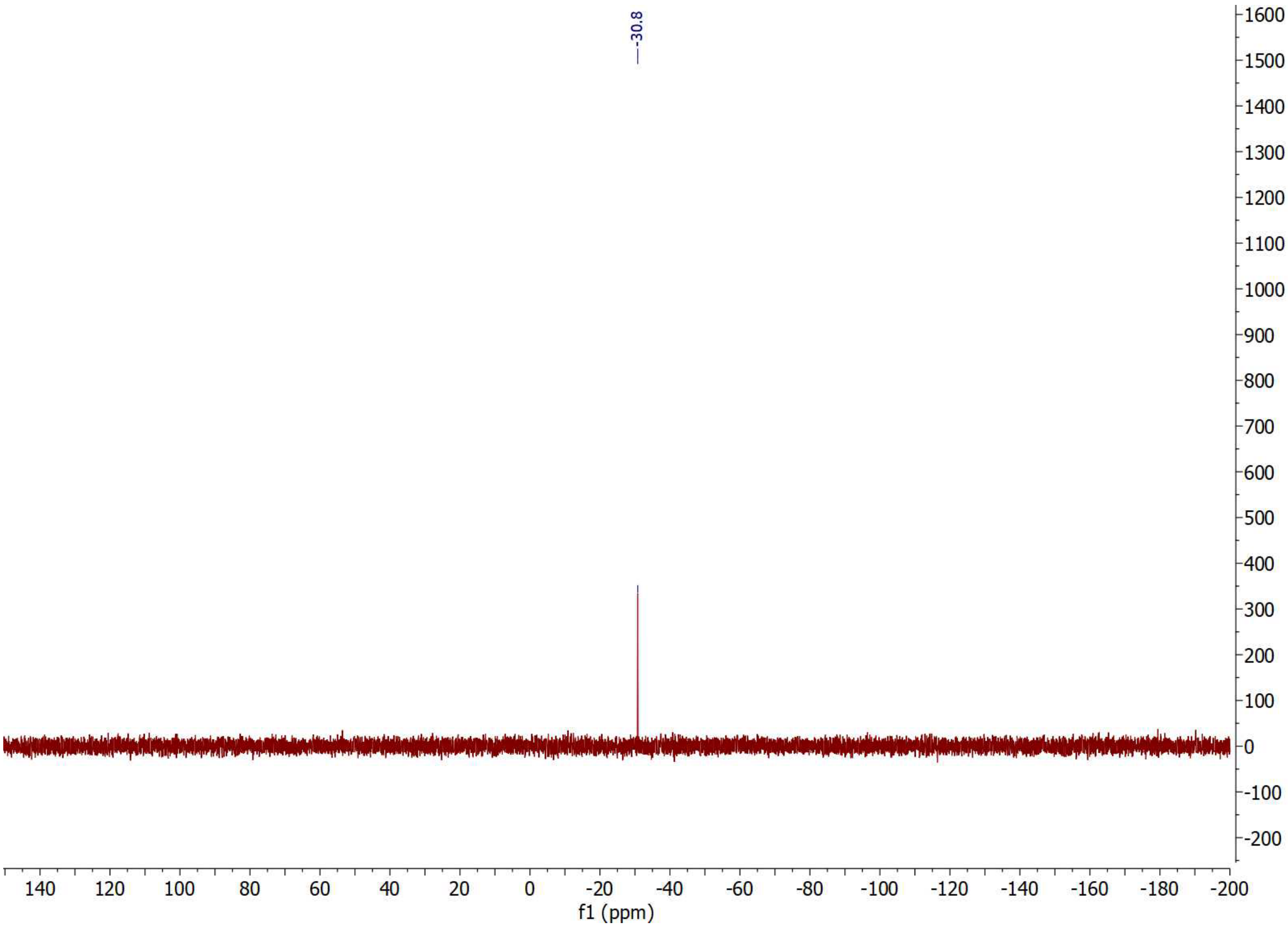

Figure S27. $^{29}Si\{^{1}H\}$-NMR spectrum of **3j**

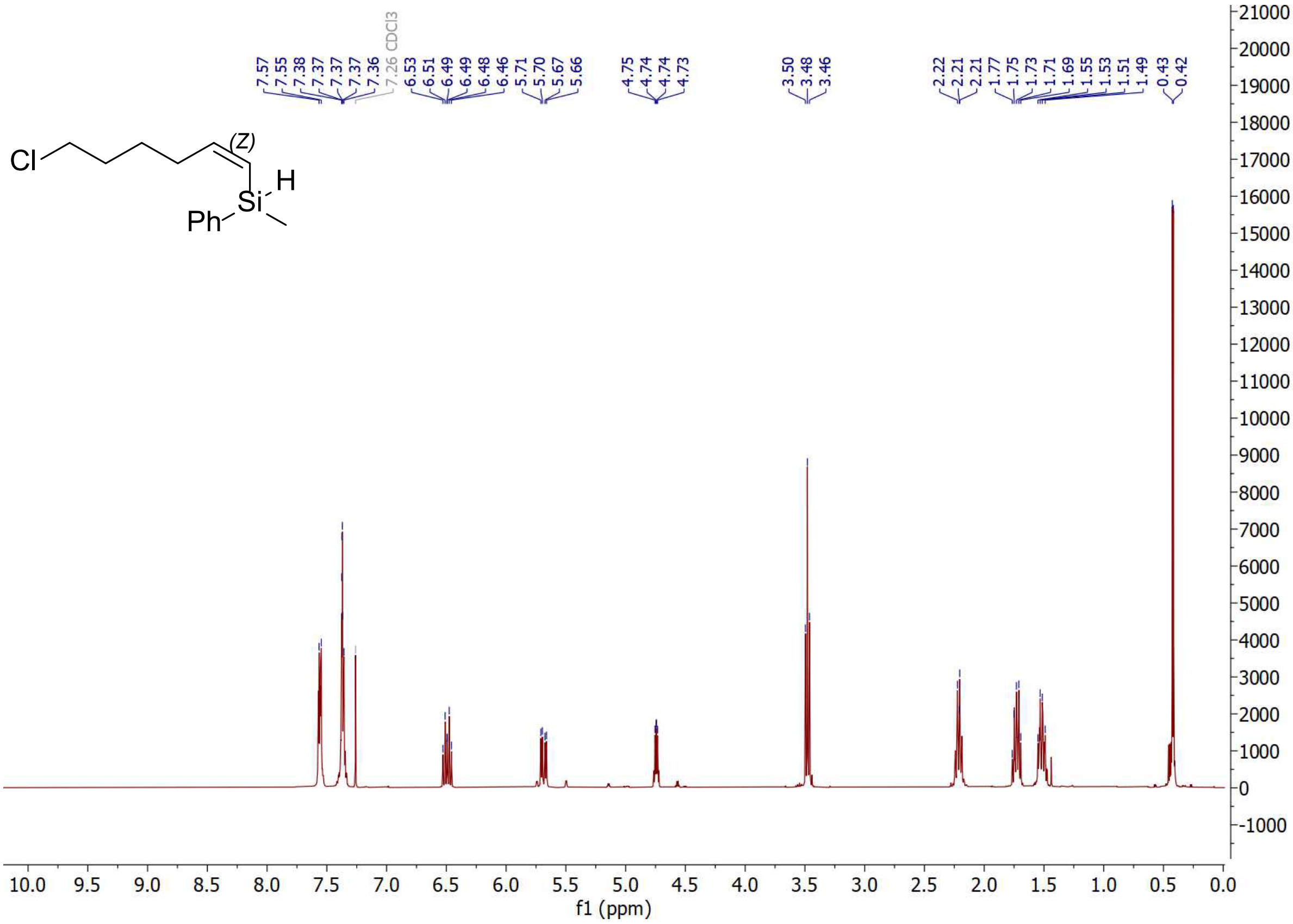

Figure S28. ^{1}H-NMR spectrum of **3k**

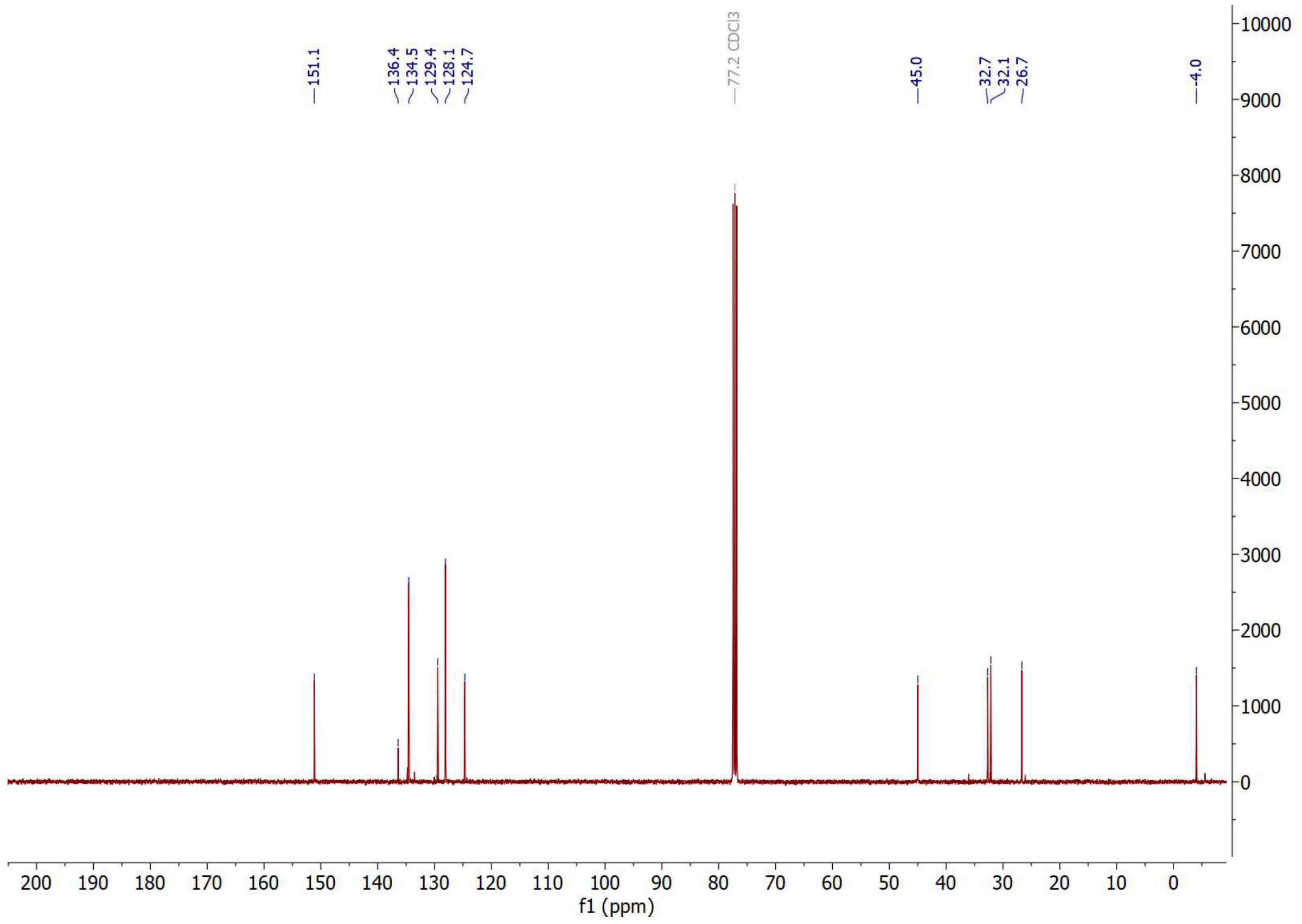

Figure S29. $^{13}C\{^1H\}$-NMR spectrum of **3k**

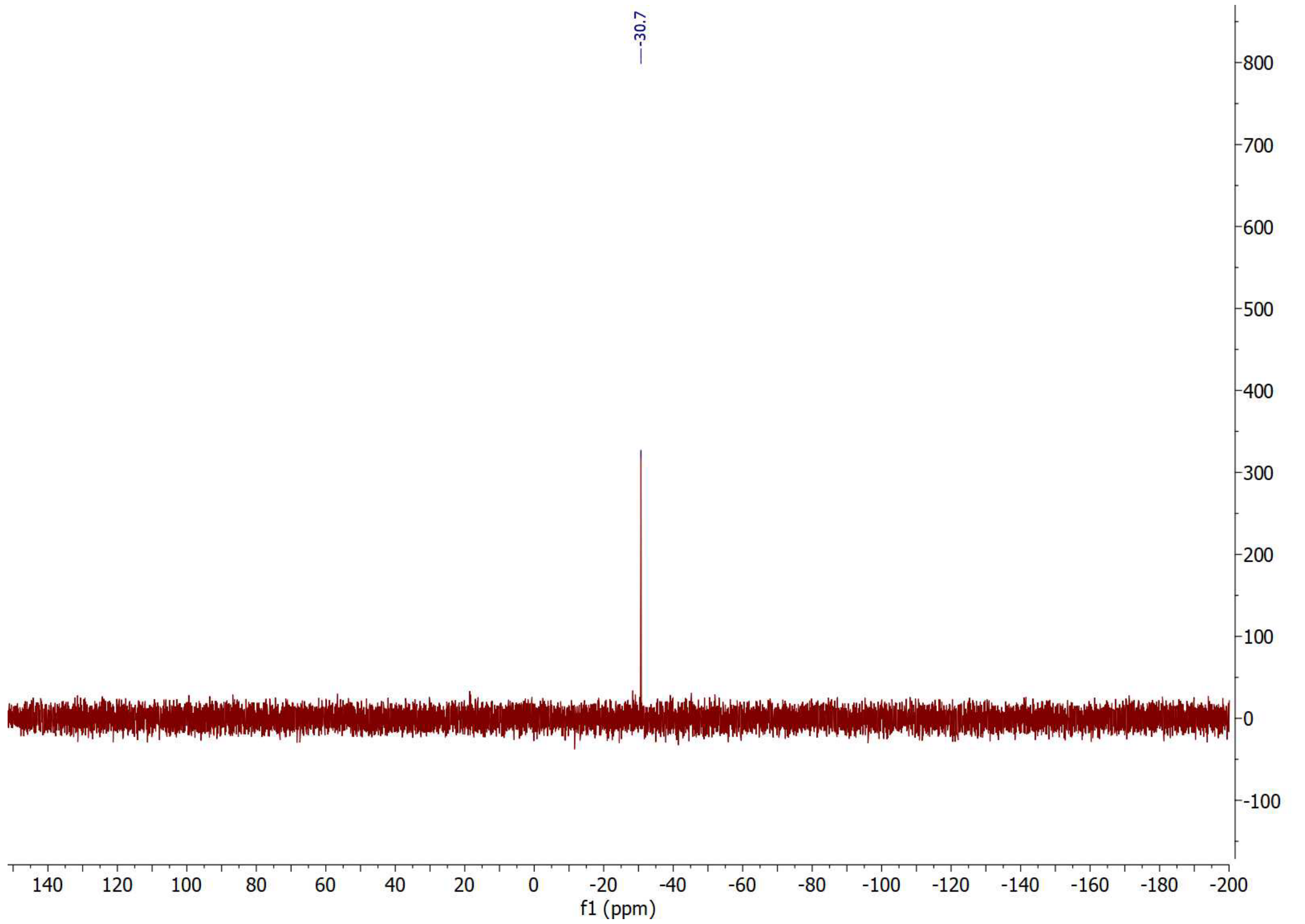

Figure S30. $^{29}Si\{^1H\}$-NMR spectrum of **3k**
